# Transcriptomic analysis of the *Myxococcus xanthus* FruA regulon, and comparative developmental transcriptomic analysis of two fruiting body forming species, *Myxococcus xanthus* and *Myxococcus stipitatus*

**DOI:** 10.1186/s12864-021-08051-w

**Published:** 2021-11-01

**Authors:** Anna L. McLoon, Max E. Boeck, Marc Bruckskotten, Alexander C. Keyel, Lotte Søgaard-Andersen

**Affiliations:** 1 Biology Department, Siena College, Loudonville, NY USA; 2 Biology Department, Regis University, Denver, CO USA; 3 Center of Medical Genetics and Human Genetics, Philipps-University, Marburg, Germany; 4 Department of Atmospheric and Environmental Sciences, University at Albany, Albany, NY USA; 5 Department of Ecophysiology, Max Planck Institute for Terrestrial Microbiology, Marburg, Germany

**Keywords:** Transcriptomics, Bacterial development, Genetics, Gene regulation, FruA

## Abstract

**Background:**

The Myxococcales are well known for their predatory and developmental social processes, and for the molecular complexity of regulation of these processes. Many species within this order have unusually large genomes compared to other bacteria, and their genomes have many genes that are unique to one specific sequenced species or strain. Here, we describe RNAseq based transcriptome analysis of the FruA regulon of *Myxococcus xanthus* and a comparative RNAseq analysis of two *Myxococcus* species, *M. xanthus* and *Myxococcus stipitatus*, as they respond to starvation and begin forming fruiting bodies.

**Results:**

We show that both species have large numbers of genes that are developmentally regulated, with over half the genome showing statistically significant changes in expression during development in each species. We also included a non-fruiting mutant of *M. xanthus* that is missing the transcriptional regulator FruA to identify the direct and indirect FruA regulon and to identify transcriptional changes that are specific to fruiting and not just the starvation response. We then identified Interpro gene ontologies and COG annotations that are significantly up- or down-regulated during development in each species. Our analyses support previous data for *M. xanthus* showing developmental upregulation of signal transduction genes, and downregulation of genes related to cell-cycle, translation, metabolism, and in some cases, DNA replication. Gene expression in *M. stipitatus* follows similar trends. Although not all specific genes show similar regulation patterns in both species, many critical developmental genes in *M. xanthus* have conserved expression patterns in *M. stipitatus*, and some groups of otherwise unstudied orthologous genes share expression patterns.

**Conclusions:**

By identifying the FruA regulon and identifying genes that are similarly and uniquely regulated in two different species, this work provides a more complete picture of transcription during *Myxococcus* development*.* We also provide an R script to allow other scientists to mine our data for genes whose expression patterns match a user-selected gene of interest.

**Supplementary Information:**

The online version contains supplementary material available at 10.1186/s12864-021-08051-w.

## Background

Bacteria have evolved several strategies that allow their survival under varied environmental conditions. Typically, these strategies involve changes in gene expression, metabolism and/or motility. However, multiple taxa of bacteria withstand periods of low moisture and nutrient availability by forming metabolically inactive heat and desiccation resistant spores. Spore formation is well-studied in the phylogenetically widely separated model species *Bacillus subtilis*, *Streptomyces coelicolor* and *Myxococcus xanthus;* however, the cellular differentiation processes resulting in spore formation is dramatically different between these three species. Spore formation in *B. subtilis* begins with rearrangements of the DNA to form an axial filament followed by an asymmetric cell division of the rod-shaped cell resulting in the formation of a large mother cell and a small forespore. It is this forespore that differentiates into the dormant, highly resistant spore, while the mother cell synthesizes many of the proteins needed to form the spore coat, then lyses [1]. In *S. coelicolor*, chains of spherical spores are formed by multiple synchronous cell divisions in aerial hyphae extending from the colony surface [2]. In *M. xanthus*, sporulating cells round up without a prior cell division inside fruiting bodies [3–5].

Despite the different differentiation processes leading to spore formation and physical differences between the spores produced by these distinct taxa, sporulation as a survival mechanism is often initiated as a social process within a multicellular community. *B. subtilis* sporulation is coupled to the formation of multicellular biofilm communities and is the culmination of biofilm formation [6–8]. *S. coelicolor* does not sporulate in liquid, but needs extensive signaling and coordination within a multicellular mycelium [2]. Similarly, in *M. xanthus*, spore formation is coupled to the formation of multicellular spore-filled fruiting bodies. Specifically, only cells that have accumulated inside a fruiting body differentiate into spherical spores, and they do so without requiring an additional cell division. Here, we begin to explore the conservation of the developmental program of the Myxococcales that allows most members of this order to withstand starvation through the formation of spore-filled fruiting bodies [9, 10].

The Myxococcales are Gram-negative bacteria that belong to the deltaproteobacteria (of note, it was recently proposed that Myxococcales would make up a separate phylum, the *Myxococcota*, with Myxococcales referring to an order under the proposed system [11]). Soil-dwelling myxobacteria are typically predatory as well as saprophytic, and most members respond to nutrient starvation with the initiation of a developmental program that culminates in the formation of spore-filled fruiting bodies. Because the predatory, saprophytic and developmental behaviors are cell density and cell contact-dependent, the Myxococcales are often referred to as social bacteria [12–14]. It is generally thought that the social coordination of sporulation in discrete fruiting bodies enables dispersal of sufficient cell numbers to allow the group to more easily resume social feeding behaviors when nutrients become available [15].

Over the past decades, much has been learned about fruiting body formation based on work on the model species *M. xanthus.* Briefly, fruiting body formation requires sufficient cells to be present on a solid surface. In response to starvation, cells change their motility behavior and coordinate their movements in a pattern distinct from the coordinated movement pattern in the presence of nutrients, ultimately leading to the formation of mound-shaped aggregates approximately 12 h after initiation of starvation in typical laboratory strains [9]. Over time, more cells accumulate in the aggregation centers; by approx. 24 h, the aggregation process is finished with the formation of nascent fruiting bodies. Over the next 48 to 96 h, the cells that have accumulated inside fruiting bodies differentiate into spores. Two additional cell fates arise during this developmental program: 1) cells that do not join the aggregates remain rod-shaped and are referred to as peripheral rods [16–18] and 2) a large fraction of cells undergo lysis [16].

At the molecular level, fruiting body formation in *M. xanthus* depends on cells being motile, signaling by the intracellular nucleotide-based second messengers (p)ppGpp and c-di-GMP as well as by five intercellular signals, referred to as the A-E signals [9, 19–21]. Moreover, fruiting body formation depends on global changes in gene expression, and multiple transcription factors have been identified that are important for fruiting body formation [22, 23]. Also, multiple two-component transduction systems and Ser/Thr kinases have been identified as important for development [24]. Two well-studied transcription factors, MrpC and FruA, that are essential for development are activated early during fruiting body formation [25–27]. FruA activation depends on the MrpC-dependent *fruA* transcription in response to starvation and the intercellular C-signal; subsequently MrpC and FruA act coordinately and are essential for the downstream gene regulatory changes responsible for fruiting body formation [25, 28–31]. Ultimately these intra- and intercellular signals and transcription factors lead to the execution of this complex genetic program. Using genetics and global transcriptional profiling, hundreds of genes have been demonstrated to be developmentally regulated at the transcriptional level, many of which are important for development [23, 32–41].

While many details have been worked out for the genetic program underlying fruiting body formation in *M. xanthus*, little is known about the molecular basis for fruiting body formation and sporulation in other species of Myxococcales. Generally, *Myxococcales* have large genomes and their fully sequenced genomes are mostly 9–16 Mb in size, the exceptions being those of *Vulgatibacter* and *Anaeromyxobacter* species, which have genomes of approximately half the size and also a corresponding simplification of lifestyle with no reports of fruiting body formation and sporulation. Fruiting body morphology varies between species including haystack shaped as in *M. xanthus*, single sporangioles on a stalk like *Myxococcus stipitatus, and* complex stalked fruiting bodies topped with multiple sporangioles in *Stigmatella aurantiaca* and *Corallococcus coralloides* [42]. Previous work based on gene content comparisons has found that several essential developmental genes in *M. xanthus* are missing from the genomes of other fruiting Myxobacteria and with an inverse correlation between number of genes conserved and phylogenetic distance, suggesting that the genetic programs for fruiting body formation vary between Myxobacteria [32]. Moreover, experimental evolution experiments with *M. xanthus* have also showed that development can be restored to a fruiting-deficient mutant using a different transcriptional program than that in WT *M. xanthus* [43]. This may suggest that our understanding of the *M. xanthus* developmental program is specific to certain laboratory conditions and may fail to include paradigms used by other Myxobacteria or those that are used by the model species *M. xanthus* in the more varied natural world.

Transcriptional changes in gene expression are essential for fruiting body formation in *M. xanthus*, but little is known about the process in closely related species. Therefore, to address the conservation of the genetic program for fruiting body formation, we mapped and compared the developmental transcriptome in two relatively closely related species. For this comparative developmental transcriptome analysis, we selected *Myxococcus stipitatus,* which forms fruiting bodies with sporangioles on a stalk, in marked contrast to the stalkless mound-shaped fruiting bodies of *M. xanthus* [42], for several reasons: 1. Its genome has been fully sequenced [44]; 2. It is sufficiently closely related to *M. xanthus* that genome synteny and orthologs can be easily identified (see below); and 3. we could induce fruiting body formation reproducibly and at a large enough scale to isolate RNA. Although the *M. stipitatus* genome has been sequenced completely, little to nothing has been experimentally determined regarding the genetic and molecular mechanisms of fruiting body formation in *M. stipitatus* [44]. Indeed, publications about this organism are essentially limited to its use as a source for the identification and characterization of diverse natural products [45–47]. Here, we use RNAseq to compare the developmental transcriptomes of *M. xanthus* and *M. stipitatus.* While we describe the substantial differences in gene expression patterns during fruiting body formation between the two species, we also demonstrate that core principles that were described in *M. xanthus* are also true for *M. stipitatus*, namely that transcriptional regulators and signal transduction mechanisms are expressed highly during development and that protein production and many metabolic processes are expressed at lower levels in developing cells.

## Results and discussion

### Comparison of genomes and genome annotations

As is true for many Myxococcales, both *M. xanthus* and *M. stipitatus* have large GC rich genomes. Although the genomes were initially sequenced in 2006 and 2013, NCBI has automatically reannotated both genomes twice using the automated Prokaryotic Genome Annotation Pipeline (PGAP), which resulted in the annotation of 7348 and 8143 annotation entries in the two genomes [44, 48–50]. Of the annotations identified in the update for *M. xanthus* strain DK1622 (NC_008095.1), 7181 of them are putative coding sequences, and 167 are various non-coding transcripts or pseudogenes (Table 1 and Additional file 23). Similarly, of the 8143 genes identified in the updated annotation for *M. stipitatus* strain DSM14675 (NC_020126.1), 7879 of them are putative coding sequences, and 264 are various non-coding transcripts or pseudogenes. For the work described here, we have analyzed protein-coding gene and pseudogene annotations, since it is possible that some pseudogenes are functional genes in which there was a sequencing error or a spontaneous mutation in the sequenced isolate.

**Table 1:** Summary of genome annotations in *M. xanthus* strain DK1622 and *M. stipitatus* strain DSM14675

	***M. xanthus***	***M. stipitatus***
**Genome size (bp)**	9,139,763	10,350,586
**%GC**	68.9	69.2
**Genes, total** (initial annotation)	7404	8129
**Genes, total** (newest annotation)	7348	8139
CDS	7181	7798
rRNA	12	9
tRNA	65	77
Miscellaneous RNA (non-coding)	4	4
Pseudogenes	86	174

**Fig. 1:**
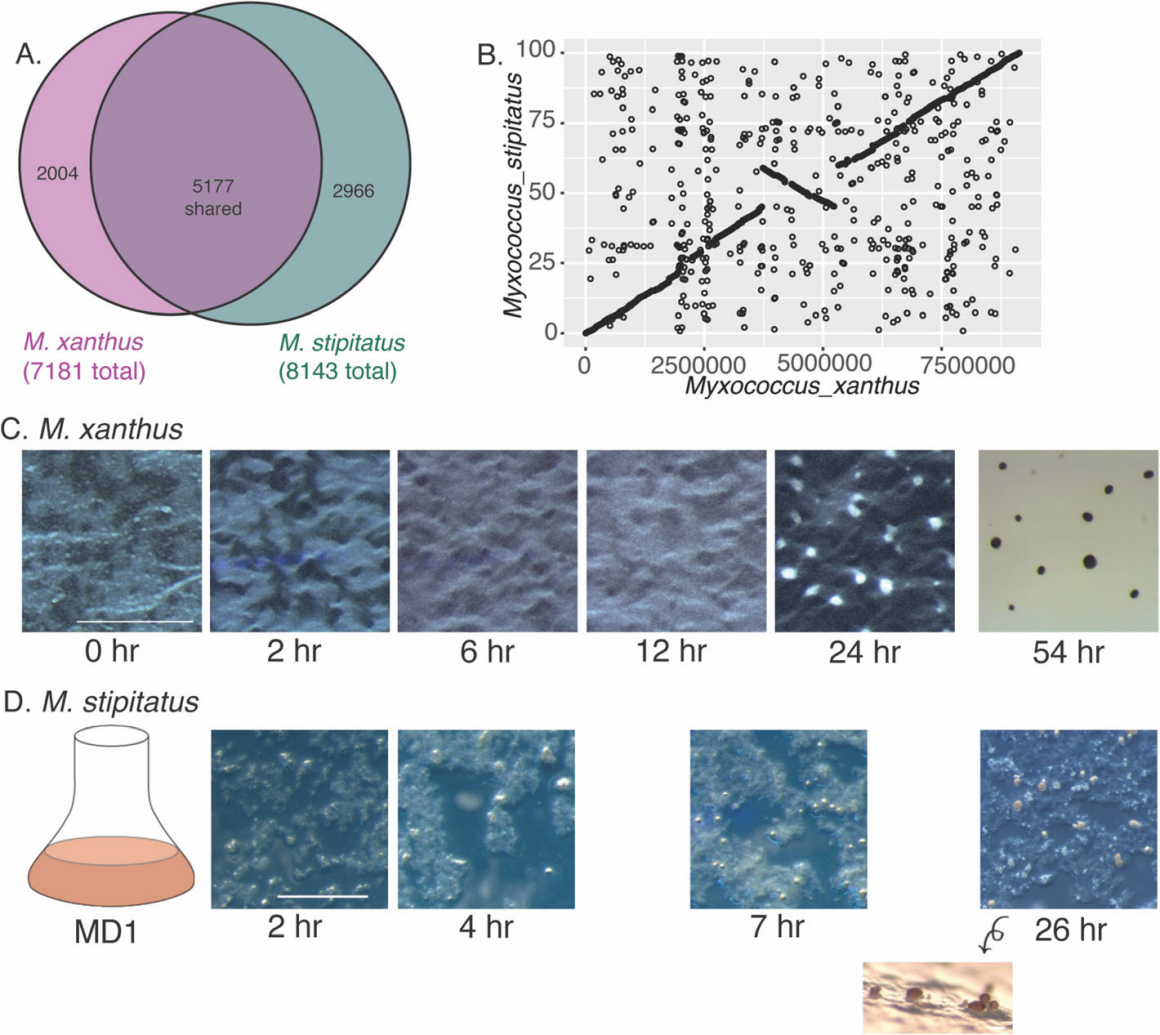
*Myxococcus* development occurs through a series of coordinated multicellular processes. **A** Genes present in each genome are represented in this Venn diagram produced using BioVenn. **B** EDGAR was used to determine synteny using reciprocal best-hit BLAST and was plotted using ggplot2. Images of developing *M. xanthus* (**C**) and *M. stipitatus* (**D**) cells at the indicated timepoints. Scale bars represent 500 μm (**C**), 1 mm (**D**), and 100 μm (side-view)

*M. xanthus* and *M. stipitatus* are closely related according to 16 s rRNA sequences, and both belong to the Cystobacterineae. Nonetheless, although many orthologous genes show high levels of conservation, for instance the *fruA* gene has an 87% identity and the protein 95% identity, there are substantial differences in gene content and genome structure. We used the EDGAR comparative genomics platform, which uses reciprocal best-hit BLAST, to identify orthologous genes in the two species [51, 52]. Five thousand one hundred seventy-seven genes are shared by the two species, which represent 72.1% of the *M. xanthus* genome and 63.6% of the *M. stipitatus* genome (Fig. 1a). Synteny of conserved genes is largely, but not perfectly, conserved between the two species (Fig. 1b).

### Gene expression and developmental dynamics in *M. xanthus*

**Fig. 2:**
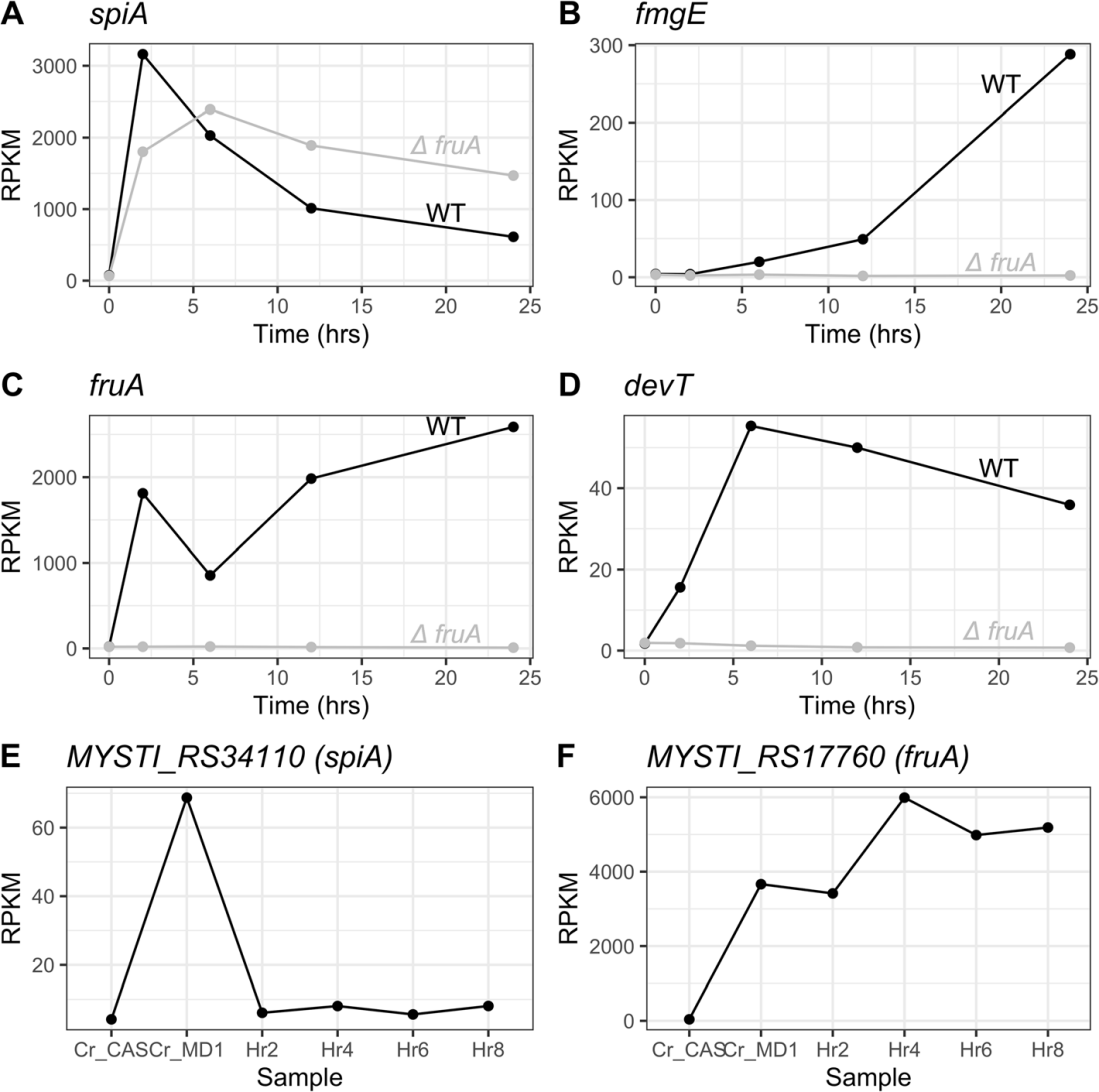
Representative known developmental genes have expected expression patterns. Average RPKM values for WT (black points) and Δ*fruA* (grey points) *M. xanthus* for genes *spiA* (MXAN_RS20760/ MXAN_4276) (**A**), *fmgE* (MXAN*_*RS16790/ MXAN_3464) (**B**), *fruA* (MXAN_RS/ MXAN_3117) (**C**), *devT* (MXAN_RS35150/ MXAN_7263) and from WT *M. stipitatus* (**E**-**F**) were calculated for each gene and timepoint from the mapped, quantified count data and were graphed using ggplot2. *M. stipitatus* “ctrl” denotes the rich medium MD1 + CAS and “ctrl_MD1” denotes the medium in which cells were cultured prior to starting fruiting assays

We isolated RNA from two biological replicates each for five time-points of WT *M. xanthus* developmental samples, corresponding to 0, 2, 6, 12 and 24 h of development in submerged culture (Fig. 1c). To this end, cells were initially grown for 24 h covered by 1% CTT on a plastic surface to form a lawn of cells. For the 0 h starvation sample, cells were directly harvested from the CTT. For the other samples, the CTT was removed and replaced with MC7 starvation buffer, and hours of development denote hours in the presence of MC7 starvation buffer. We selected these time points as representative of distinct stages during the transition to and early stages of fruiting body formation that are also prior to extensive sporulation, since other work has focused extensively on the sporulation transcriptome [34, 53]. At each time point, we harvested all cells adhered to the plastic surface, so the observed expression patterns are not specific to any cell fate, and include cells that would have sporulated, lysed, or remained as peripheral rods. After isolation of total RNA, rRNA was depleted before sequencing. As a quality control of the RNAseq data, we observed that the expression patterns of four developmentally regulated genes matched those from previous reports, with *spiA*, *fruA*, and *devT* showing substantial early upregulation, and *fmgE* demonstrating transcription later during development (Fig. 2a-d) [26, 34, 35, 54]. We also looked more generally at expression in 95 previously identified genes specifically important for fruiting body formation and sporulation [32]. While some show dramatic increases in expression during development, many other genes show modest or less predictable expression patterns (Additional file 1).

**Fig. 3:**
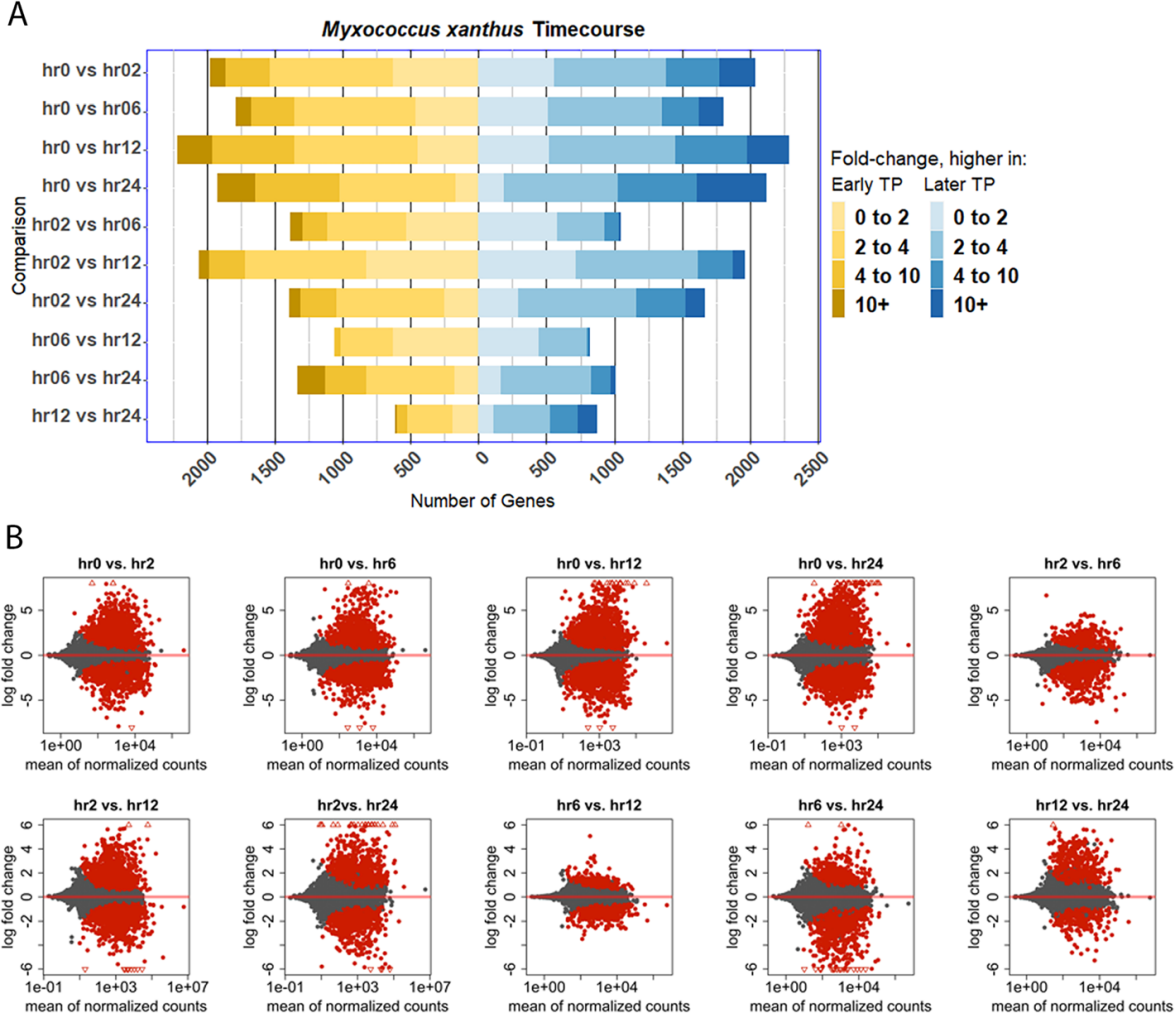
Many genes are developmentally regulated during *M. xanthus* fruiting body formation. Reads generated from WT *M. xanthus* were mapped and assigned to genes using BWA to the most recent reference genome annotations, and then the changes in gene expression during the commitment to development were quantified using HTseq and DEseq, comparing earlier with later timepoints in all pairwise combinations. Bar graphs represent numbers of genes with higher expression in the earlier timepoint (yellow) or the later timepoint (blue) for each pairwise comparison, and color intensity represents the observed log2fold change in expression. MA-plots (**B**) were generated for each pairwise comparison between timepoints using the R code provided with DEseq, including apeglm transformation to reduce variance; red points are those with an adjusted *P*-value of ≤0.01 and triangles represent genes with a log2 fold change that exeeds the maximum value on the Y-axis

Next, we carried out quantitative expression analysis using HTseq and DESeq to quantify reads that map to each gene and then used these quantifications to compare expression in each vegetative or developmental timepoint with each other in all pairwise comparisons [55, 56]. This demonstrated that large numbers of genes are regulated during development, and this includes genes with low mean levels of transcription and genes with high levels of transcription (Fig. 3). Based on DESeq, over 4000 genes show statistically significant regulation during fruiting body formation, and many of those genes show very large changes in expression levels, including hundreds of genes showing a 10-fold or greater change in expression between timepoints (Fig. 3a). Differentially expressed genes are fairly evenly split between those with higher expression levels in vegetative conditions or earlier fruiting timepoints (yellow in Fig. 3a) or with higher expression levels in developing cells or in later developmental timepoints, although slightly more genes have increased developmental expression (blue in Fig. 3a). Notably, the gene expression differences are most dramatic between non-fruiting cells and any of the fruiting timepoints. In *M. xanthus*, there are still many genes whose expression levels change between developmental timepoints (Fig. 3), although a larger variability between biological replicates at the later timepoints, especially 24 h, has increased the adjusted *p*-value for the expression changes involving these timepoints (Additional files 2 and 24). Adjusted *p*-values here and in subsequent analyses refer to those generated by the default DEseq2 settings, and represent adjustment using a Benjamini-Hochberg procedure [55]. Interestingly, a greater variation between replicates in 24 h samples was previously observed, suggesting this difference has a biological explanation that explains the shorter height of the volcano plots, despite the large expression changes [34].

### Identification of the FruA regulon

Since fruiting body formation is triggered by nutrient deprivation, it is likely that many of the genes up- or down-regulated during fruiting body formation are responding generally to the altered nutrient availability. To identify up- and down-regulated genes that are more directly involved in fruiting body formation, we compared gene expression between WT and Δ*fruA* cells by isolating RNA from Δ*fruA* cells grown under the same conditions as WT, and harvested at the same developmental timepoints. FruA is a transcription factor that is specifically synthesized during development and required at very early timepoints for development in *M. xanthus* [25], and is conserved in sequenced members of the Cystobacterineae. Thus, genes that are differentially expressed between the WT and Δ*fruA* cells are directly or indirectly under FruA control and their regulation is specific to this developmental process. While the developmental gene *spiA* does not require FruA for activation (Fig. 2a), other key developmental markers fail to become activated in the Δ*fruA* strain (Fig. 2b-d).

**Fig. 4:**
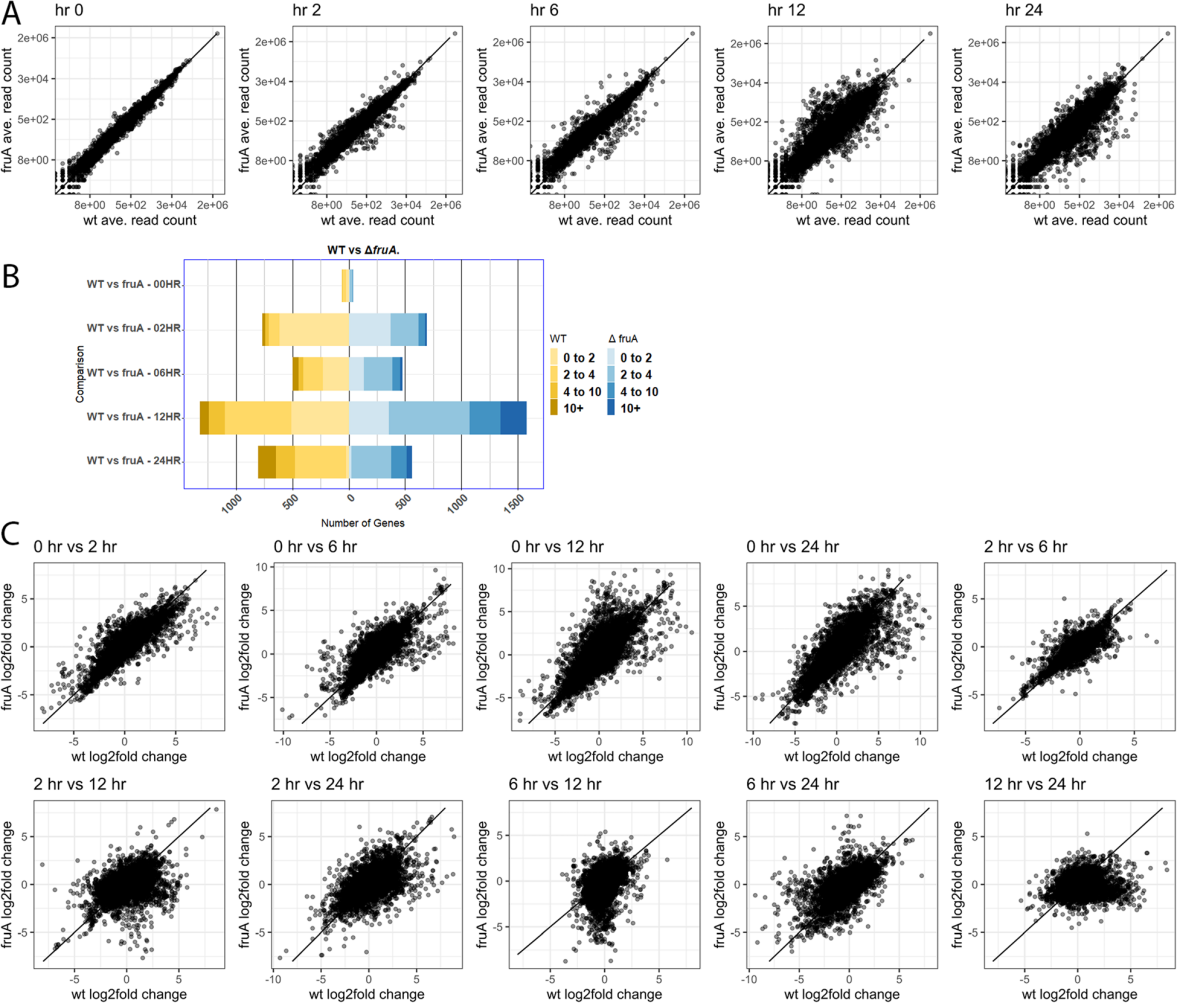
The FruA regulon is extensive, but does not include all developmentally regulated genes. RNA from a Δ*fruA* strain was isolated and sequenced. The reads per gene for WT and Δ*fruA* cells were averaged over the two biological replicates and normalized to library size, and these normalized, average read counts are compared in scatter plots (**A**). The read counts for WT and Δ*fruA* for each time point were also compared using HTseq and DEseq, and are presented as bar graphs showing the log2 fold change of genes statistically significantly up-regulated in WT (yellow) and in Δ*fruA* (blue) (**B**). HTseq and DEseq were used to generate all pairwise time comparisons for WT and Δ*fruA* separately, and the log_2_ fold changes were extracted from the DEseq outputs. Scatter plots (**C**) represent a comparison between the log_2_ fold changes for WT on the x-axis, and Δ*fruA* on the y-axis. Genes that are more highly expressed in WT cells are represented by points below or to the right of the trend line, while genes that are expressed at higher levels in Δ*fruA* cells are above or to the left the trend line

We first compared the expression levels of genes at each developmental timepoint by plotting the average number of reads mapped to each gene in WT vs. in Δ*fruA* cells at each time point (Fig. 4a). We then used HTseq and Deseq to compare read count values between the two strains for each time point and quantified the genes that are higher in WT (yellow) or Δ*fruA* (blue) cells at each timepoint (Fig. 4b and Additional file 25). As expected, relatively few genes show expression differences between vegetative WT and Δ*fruA* cells, as this is a time when *fruA* is not expressed. Specifically, at t = 0 h, only 8 genes have greater than 2 log_2_ fold changes in expression and an adjusted *P*-value smaller than 0.05. Over 1000 genes show statistically significant differences in expression between the WT and Δ*fruA* cells at one or more later timepoints, although the effects are modest for most regulated genes. Interestingly, the effects of FruA are not limited to activation of genes, as there are genes whose expression is substantially higher in Δ*fruA* cells, compared to WT (blue in Fig. 4b). We also see that FruA regulates expression of genes with varied absolute levels of gene expression, including modestly and highly expressed genes (Fig. 4a).

Besides comparing expression levels of genes at specific time points between WT and Δ*fruA* cells, we were interested in comparing expression changes between WT vs. Δ*fruA* cells. To that end, we extracted the log_2_ fold-change from separate Deseq analyses of each pairwise combination of time points for a single strain, and then we generated scatter plots comparing the Δ*fruA* fold changes with those of WT cells (Fig. 4c and Additional files 23 and 26). In all pairwise comparisons of WT cells with all the Δ*fruA* mutant cells, it is evident that there are many genes that show reduced or increased expression in the Δ*fruA* mutant. We conclude that FruA influences gene expression, directly or indirectly, positively as well as negatively. We note, however, that the transcriptional regulator MrpC is highly transcribed in our Δ*fruA* cells, so some gene regulation events may still be occurring that are necessary and specific to fruiting but not sufficient in the absence of FruA. Nonetheless, these data suggest a potential new paradigm for FruA’s activity in regulating fruiting body formation. It is generally assumed that FruA is activated by MrpC to aid in activating the developmental pathway, and recent work suggests that MrpC is transcribed proportionally to nutrient availability [57]. In contrast, these results suggest that FruA could have an additional role. It may not just activate genes needed for development, but may also be necessary for decreases in expression of genes that are otherwise highly expressed in a non-fruiting strain under conditions where development should occur. One clear example is prolonged elevated expression of the gene SpiA that is typically only expressed early during development (Fig. 2). As the two strains were not harvested in parallel, batch effects could have influenced gene expression and be responsible for some of the observed variation [58]; however, vegetative gene expression in WT and Δ*fruA* cells was well matched. Thus, we hypothesize that rather than merely acting coordinately with MrpC to activate genes, FruA may additionally act to fine-tune and dampen gene activation initiated by MrpC in direct response to starvation, and this could contribute to FruA’s necessary role in fruiting body formation.

### Specific protein domains and COG annotation categories are enriched in developmentally regulated genes

We next sought to identify patterns of gene expression in the WT and Δ*fruA* cells. As we wanted to limit our analysis to genes whose expression changes are most likely to be biologically relevant, we first filtered out genes with low expression then measured the variability of expression of the remaining genes between sample types. We chose the 2000 genes with the greatest overall variability in expression within the whole *M. xanthus* dataset for clustering and further analysis. These genes were 1 or more standard deviations above the mean for variability of expression.

**Fig. 5:**
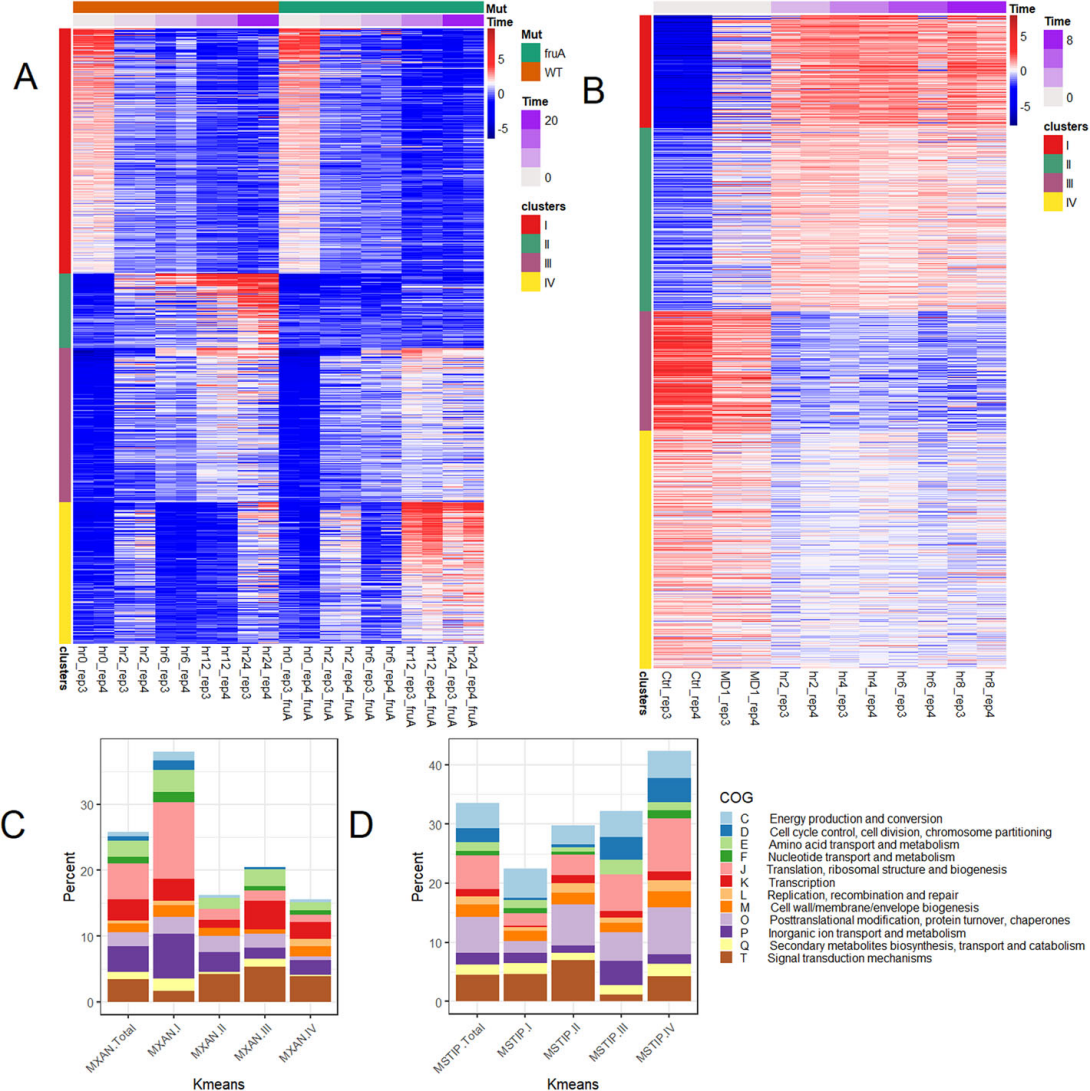
Kmeans heatmap and COG enrichment for each Kmeans cluster for *M. xanthus* WT and Δ*fruA*, and for *M. stipitatus*. **A** Kmeans heatmap was generated for the 2000 *M. xanthus* genes clustered into 4 clusters. Genes were clustered based on similar expression patterns, and samples were placed in order based on time after fruiting induction and mutational status. More red color designates higher expression for a given sample while blue designates lower expression. Mutational status and time after fruiting induction are shown on top. **B** Kmeans heatmap for 2000 *M. stipitatus* genes clustered into 4 clusters. Genes were clustered based on similar expression patterns, samples were placed in order based on time after fruiting induction and mutational status. More red color designates higher expression for a given sample while blue designates lower expression. Time after fruiting induction is shown on top. **C** Percent of *M. xanthus* genes from each Kmeans cluster falling into each COG category. **D** Percent of *M. stipitatus* genes from each Kmeans cluster falling into each COG category

We then carried out Kmeans clustering, which identified four clusters of gene expression for *M. xanthus,* clusters I, II, III, and IV (Fig. 5a, Additional file 29). Optimal cluster numbers were determined heuristically by choosing the number of clusters such that the within-cluster sum of squares (WSS) did not improve by adding any more clusters.

Cluster I is composed of 797 genes that are upregulated in the 0 h time point and decrease expression during development independently of FruA. Cluster II is composed of 240 genes that are not expressed early and become more highly transcribed as development proceeds in a FruA-dependent manner. Cluster III is composed of 502 genes that are not expressed early and are expressed later during development and are FruA independent. Cluster IV is composed of 461 genes that are expressed at low levels early during development, and then turned on at the later developmental timepoints; however, these genes are more highly expressed at 12 and 24 h in the Δ*fruA* mutant. These are genes that are developmentally repressed by FruA-dependent mechanisms (Fig. 5a, Additional file 29).

We next identified the COG annotations of genes to look for functional categories enriched in each Kmeans cluster (Fig. 5c and Additional file 28). Cluster I, vegetative genes that are down-regulated during development independently of FruA, is enriched for genes involved in energy production and conversion (pale blue), cell cycle control (dark blue), translation (pink) and inorganic ion transport and metabolism (dark purple). In contrast, these functional categories are underrepresented in all three developmental gene clusters (Fig. 5) including FruA-dependent (cluster II and IV) and FruA-independent (cluster III) genes. A previously published developmental transcriptome identified selected metabolic genes downregulated during fruiting body formation, and we see that their observation is more generally true [34]. In contrast, genes assigned to COG category T (signal transduction mechanisms) are enriched in all three developmental clusters, and genes assigned to COG category K (transcription) are enriched in cluster III, the FruA-independent developmentally upregulated genes.

As COG annotations have limitations, we also sought to identify protein domains and gene families that are more likely to be developmentally regulated. We therefore used the web application iDEP to identify protein domains from the Interpro and pfam databases that are enriched at specific timepoints or in WT or mutant cells [59, 60]. This application is based on the Generally Applicable Gene-set Enrichment method (GAGE), which uses biological pathway data and expression data to identify protein domains that are enriched in up- and down-regulated genes identified by quantitative, differential expression analysis [55, 61].

For *M xanthus*, we carried out the analysis using the DEseq output for comparisons between vegetative cells and each developmental timepoint, and also included comparisons between WT and Δ*fruA* cells at 2 h, 12 h and 24 h timepoints. Many protein domains related to signal transduction mechanisms are clearly upregulated in developing *M. xanthus* cells, including multiple Interpro categories related to protein kinases (Additional file 4). These protein domains are not, however, identified as enriched when comparing WT and Δ*fruA* cells, suggesting that upregulation of these kinases is not FruA dependent. Polyketide synthase genes are also enriched and appear to require FruA as their expression is higher in WT samples. One other pattern of note is the developmental downregulation of several Interpro domains for Beta-ketoacyl synthases and acyl-transferases. As previous work has identified that beta-oxidation of fatty acids is important for *M. xanthus* development, we speculate that these fatty acid synthesis mechanisms are downregulated so as not to interfere with fatty acid metabolism [62]. Results are largely similar when using the pfam protein domain annotations (Additional file 5).

### Developmental transcriptome for a second Myxococcus species

While much work has focused on the developmental pathways of *M. xanthus*, comparative work can determine if those pathways are unique to one species or may be more generally conserved. We tested several related species for fruiting body formation, and were able to develop a reproducible method to induce fruiting body formation in *Myxococcus stipitatus* at a sufficiently large scale to harvest RNA and have fruiting body formation well synchronized across the plates. Initially, we attempted to replicate the method for fruiting body induction that we determined was best for *M. xanthus,* induction in submerged culture, but attempts to use a similar technique with *M. stipitatus* were unsuccessful. However, we were able to induce fruiting body formation in *M. stipitatus* by concentrating cells grown in a modified MD1 medium and plating them on starvation plates. Fruiting body formation occurs more rapidly in this system, with aggregates appearing between 6 and 8 h after plating, and mature fruiting bodies present by 24 h (Fig. 1d). Thus, to approximate the equivalent stages to those harvested for *M. xanthus*, we isolated RNA from cells grown in MD1 liquid suspension (considered the 0 h sample), and from developmental samples at 2, 4, 6, and 8 h after inducing fruiting body formation in *M. stipitatus*. In contrast to *M. xanthus,* where FruA is undetectable in Western blots of vegetative samples, FruA could already be robustly detected using antibodies to *M. xanthus* FruA by Western blot of an *M. stipitatus* MD1 (0 h) culture, suggesting some developmental genes are already expressed under these conditions (Additional file 6). Therefore, at the same time as we harvested the MD1 developmental sample, we also isolated RNA from a control culture of MD1 supplemented to 1% casitone (MD1 + CAS), in which FruA could not be detected (Additional file 6). From here on, we refer to this sample as the “Control” (Ctrl) sample. All RNA samples were then sequenced by Illumina HiSeq RNAseq, and the sequences were analyzed using BWA for alignments and HTseq and DEseq for quantification and statistical comparison of expression, respectively. While not all fruiting genes that have been identified in *M. xanthus* are present in the genome of *M. stipitatus*, *spiA* and *fruA* show similar expression patterns (Fig. 2e-f). Within the broader set of identified fruiting genes, many more also show conserved expression in the two species, including over 15 genes whose expression increases developmentally (Additional file 1). This includes some regulatory genes whose products have significant roles in the early commitment to fruiting body formation such as *mrpC*, and also the *nfsA-H* spore polysaccharide biosynthesis locus.

**Fig. 6:**
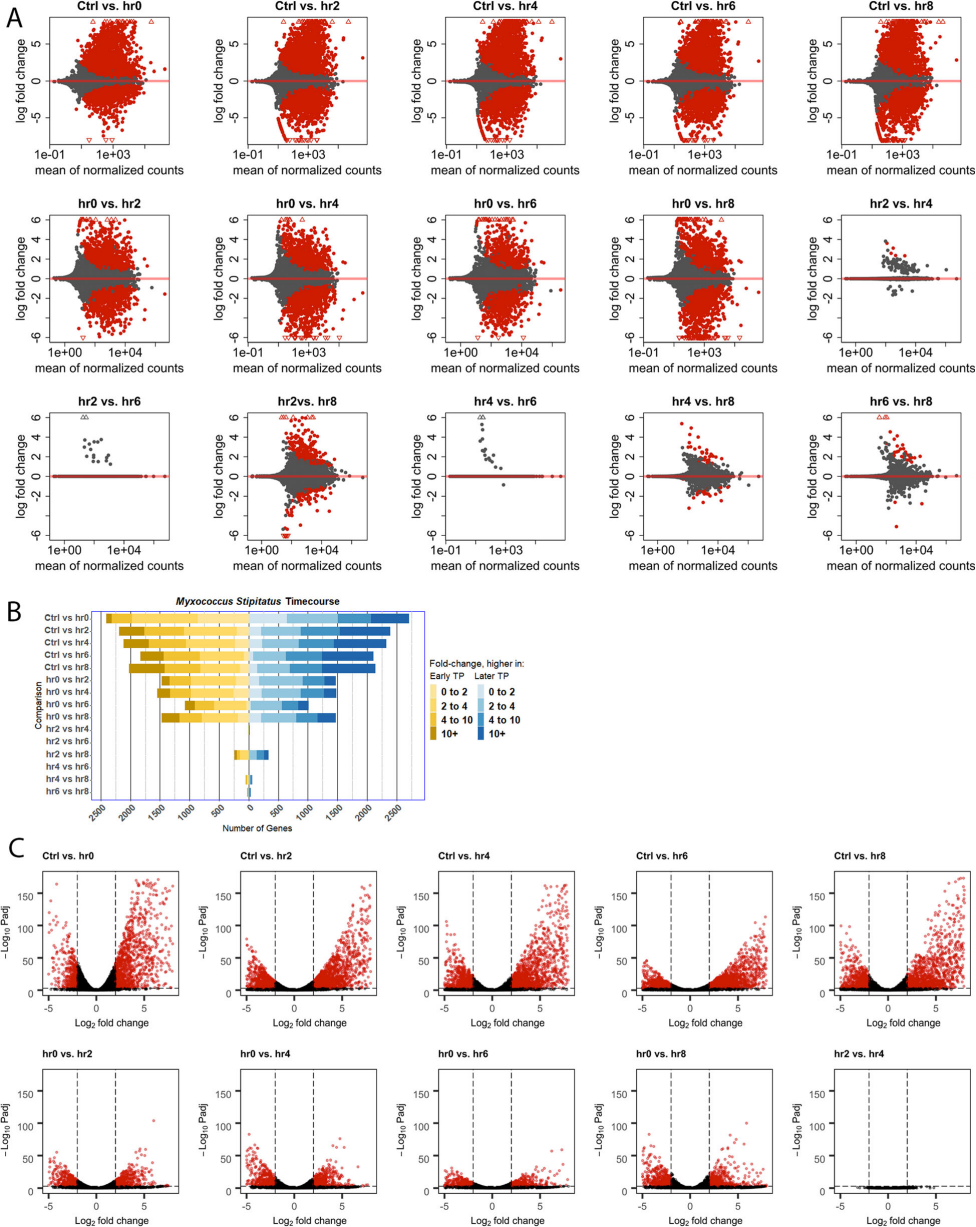
Many genes are differentially regulated between *M. stipitatus* cells grown in ctrl and MD1 media. RNA from *M. stipitatus* was isolated and was sequenced and analyzed as described for *M. xanthus*. **A** MAplots were generated using DEseq and data were apeglm transformed. Red points are those with an adjusted *P*-value of ≤0.01, and triangles represent genes with log2 fold changes greater than the values on the Y axis. **B** Bar graphs indicate the numbers of genes that show statistically significantly increased expression in the earlier timepoint or control condition (yellow) and increased expression at the later timepoint (blue) with color intensity representing the degree of regulation based on log2 fold changes. Volcano plots (**C**) were generated for using the “Enhanced volcano” R package, and points in red have log2 fold changes of ≤ − 2 or ≥ 2, and *p*-values of ≤1 × 10^− 3^

In *M. stipitatus*, gene expression as measured by read counts spans 4 orders of magnitude, as illustrated by MA plots, although many genes show low levels of expression (Fig. 6a). When we compared timepoints, over 4000 genes showed statistically significant expression changes between the vegetative control MD1 + CAS (Ctrl) sample and the 0 h MD1 sample (Fig. 6b). While fairly equivalent numbers of genes are expressed more highly in the Ctrl (2403 genes) and in the 0 h samples (2715 genes), slightly more genes are expressed over 10-fold more highly in the 0 h samples (647 genes) compared to the Ctrl (89 genes), and that holds true for comparisons between Ctrl and the later developmental samples as well, illustrating that many of the genes that are expressed at higher levels during development are expressed at dramatically higher levels. Similarly, > 3000 genes showed differential expression in developing cells compared to the 0 h sample, although this is lower than the number of genes that change between the Ctrl and 0 h samples, suggesting that many of the genes that are differentially regulated in the media that primes the cells to fruit don’t change their expression further. Interestingly, and in contrast to *M. xanthus*, in *M. stipitatus*, the gene expression changes between developmental timepoints are much less dramatic, and in fact few genes show significant changes in expression between timepoints after plating on starvation plates (Fig. 6). It is unclear if these gene regulation patterns in *M. stipitatus* are due to a lower percentage of cells actively forming fruiting bodies, or because the cells in the MD1 culture (0 h) are already transcriptionally poised to enter fruiting body formation, and thus fewer transcriptional changes are necessary.

The Interpro enrichments of developing *M. stipitatus* represent diverse protein families (Additional file 4). We do note that several DNA-related functional domains are downregulated, including 36 proteins containing a Ku70/80 beta-barrel domain, 380 DNA polymerase lambda lyase domain containing proteins, and 20 DNA primase catalytic core N terminal domain containing proteins. Since cell-cycle and DNA replication related genes are were also underrepresented in the *M. xanthus* developmental gene clusters, this suggests down-regulation of DNA replication is a conserved developmental process. Interestingly, there is enrichment of a putative “fatty acid synthesis PlsX protein” and possible glycolysis/TCA cycle related proteins including “succinate dehydrogenase/fumarate reductase” and “phosphofructokinase superfamily” proteins. This raises the possibility that fatty acid catabolism may not be as essential during fruiting body formation in *M. stipitatus*, compared to what has been described in *M. xanthus* [62]. However, the functional annotations of the *M. stipitatus* genome are less robust and more genes have no assigned function, so it seems likely that a better genome annotation could increase the practicability of this analysis.

We also carried out K-means clustering of the *M. stipitatus* data and identified four clusters, I-IV, that represent, in order: cluster l: three hundred forty-three genes with highly increased expression during development, cluster II: five hundred sixty-one developmental genes with less dramatic expression changes than genes in cluster I, cluster III: three hundred sixty-seven vegetative genes with high expression in the CTRL sample and reduced expression during development, and cluster IV: seven hundred twenty-nine vegetative genes with lower expression in the CTRL and reduced expression during development, but with generally less dramatic expression changes than genes in cluster III (Fig. 5b, Additional file 30). We also looked at enrichment of COG annotations in each cluster (Fig. 5d, Additional file 28), and found that the two developmental gene clusters (I and II) contain fewer genes that are annotated as playing a role in cell cycle control (dark blue), translation (pink) and inorganic ion transport and metabolism (dark purple). Genes assigned to the signal transduction mechanisms category (brown) are enriched in Cluster II, developmental genes with relatively lower overall expression levels, and are underrepresented in Cluster III, which are vegetative genes with relatively high expression levels. This matches expectations based on the model species *M. xanthus*, in which increases in gene regulation and decreases in protein production and cell growth are essential for development, supporting that these are likely universal patterns.

### Correlation between *M. xanthus* and *M. stipitatus* gene expression levels and expression dynamics

**Fig. 7:**
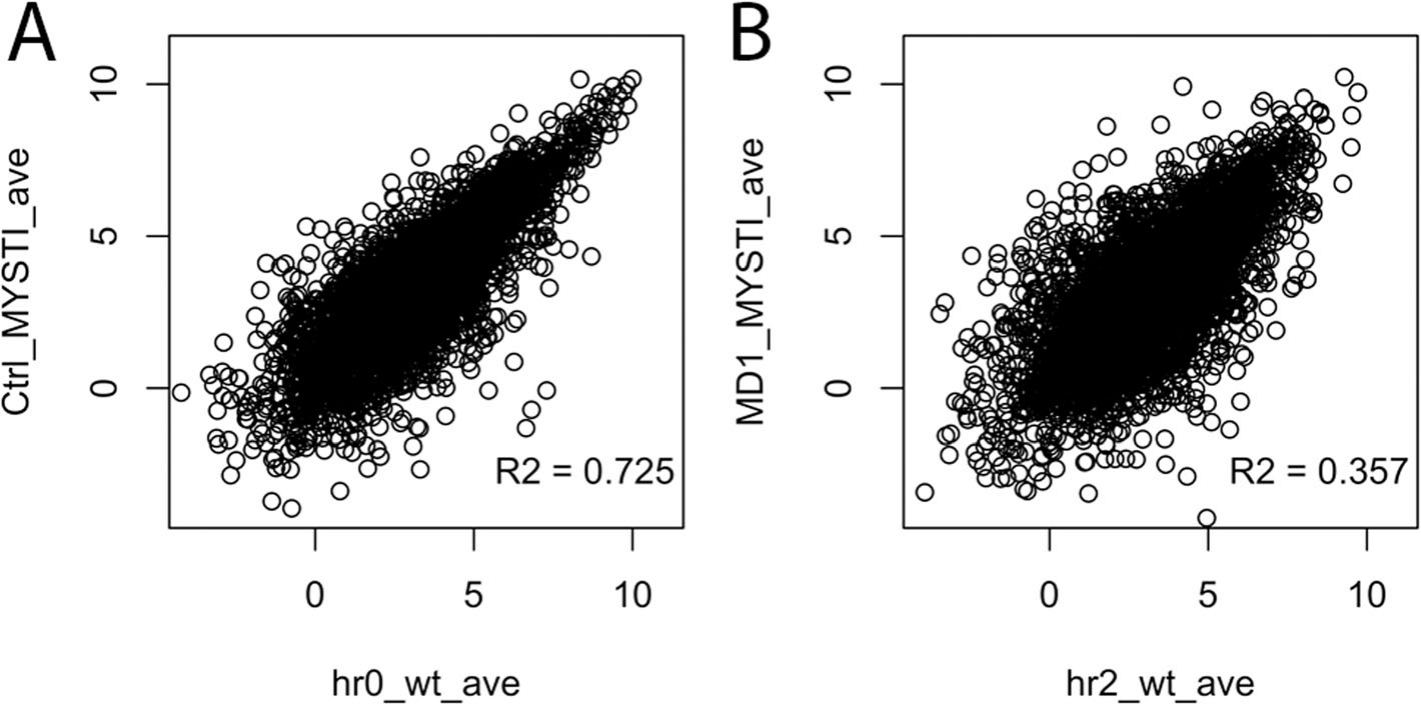
Core genes shared by *M. xanthus* and *M. stipitatus* show correlated expression levels under vegetative conditions. For each sample, read counts generated by HTseq were converted to fragments per million reads, and were averaged over the biological replicates from each strain and timepoint or condition

Although similar percentages of the genes present in *M. xanthus* and *M. stipitatus* are regulated during the course of fruiting body formation in the two species, we next examined whether orthologous genes were expressed at similar levels and were similarly regulated in the two species. We first looked at correlations between normalized gene expression levels at each timepoint in both of the two species for all 5177 genes shared between the two species, by plotting averaged RPKM values for each gene in each species. There is reasonable direct correlation in gene expression between rich medium vegetative samples with an R^2^ of 0.725 (Fig. 7a). There is also some correlation overall between the *M. xanthus* 2 h timepoint and *M. stipitatus* MD1 sample, which seems consistent with the elevated FruA levels in both samples, and supports our interpretation that the MD1 culture of *M. stipitatus* physiologically and transcriptionally resembles early fruiting body formation in *M. xanthus* (Fig. 7b). We also looked at all other pairwise comparisons of samples, and there is substantially lower correlation between all other timepoints of fruiting samples of the two species (Additional file 7).

This suggests that the fruiting assay developed for *M. stipitatus* is not the perfect biological equivalent to the assays used for *M. xanthus*. We speculate that *M. stipitatus* could require a gradual depletion of nutrients, but not direct cell-cell contact, to activate key fruiting genes and thus trigger the developmental program for fruiting body formation, which happens during overnight incubation in MD1. This could explain the rate of correlation between the MD1 *M. stipitatus* samples and the 2 h *M. xanthus* samples, as both groups of cells are experiencing reduced nutrients, but the physical proximity of cells and ability of cells to form cell-cell contacts is different in a suspension broth compared to a plate surface. Indeed, *csgA*, the developmentally induced contact-dependent C-signal of *M. xanthus*, is expressed at higher levels in MD1 compared to MD1-CAS, further suggesting that some of the genetic pathways necessary for fruiting body induction in *M. xanthus* can be activated in MD1 broth culture in *M. stipitatus*. Additionally for the *M. stipitatus* samples harvested off of starvation plates, perhaps because relatively few plated *M. stipitatus* cells are contributing to fruiting body formation using this protocol, much of the specific signal is overshadowed. While methodological differences explain some of the variation, these data likely also indicate that some of the transcriptional regulation during fruiting body formation is species specific, as well as condition dependent.

**Fig. 8:**
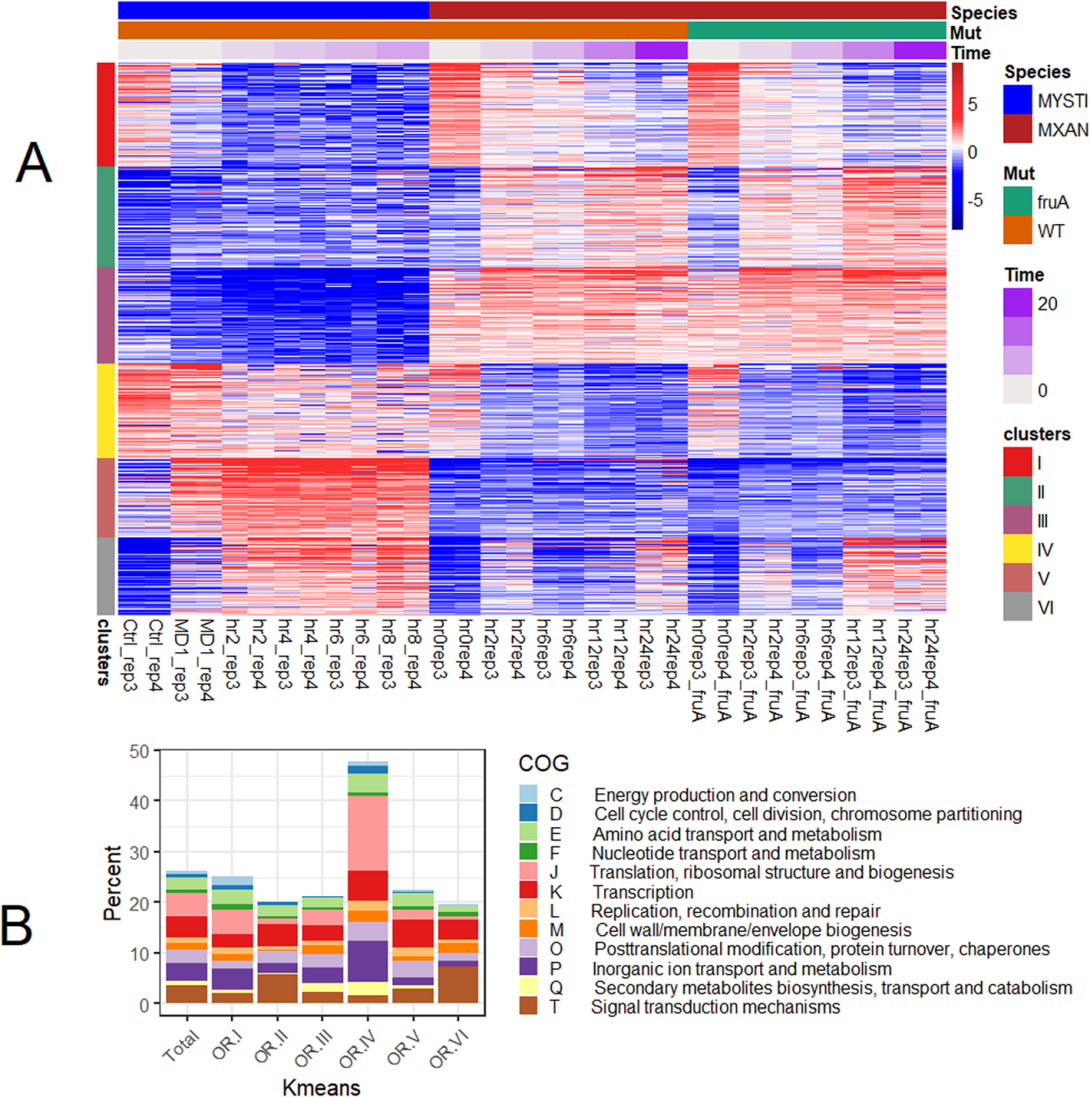
Kmeans heatmap and COG enrichment for each Kmeans cluster for orthologous genes in *M. xanthus* and *M. stipitatus*. **A** Kmeans heatmap for 2000 genes with orthologues in both *M. xanthus* and *M. stipitatus* using 6 clusters. Genes were clustered based on similar expression patterns, samples were placed in order based first on species, then on time after fruiting induction and finally mutational status as indicated by upper colored bars. For each sample timepoint, more intense red color designates higher expression for a given sample while blue designates lower expression. **B** Percent of orthologous genes from each Kmeans cluster falling into each COG category based on *M. xanthus* COG designation

Despite an overall low correspondence in developmental expression profiles between *M. xanthus* and *M. stipitatus* across all genes (Fig. 7 and Additional file 7), there are nonetheless conserved genes whose expression patterns are conserved. To specifically identify these genes, we identified 2000 orthologs with the greatest variance in expression levels in the two species, and carried out K-means clustering, using 6 clusters (Fig. 8 and Additional file 31). These six clusters have gene expression that falls into the following general patterns: cluster I contains 447 genes that generally are transcribed prior to fruiting induction regardless of species or FruA status, are downregulated during development, and are down-regulated to a more significant level in *M. stipitatus*. Cluster II contains 441 genes that are highly transcribed in *M. xanthus* after fruiting induction, but independent of FruA status, and that show minor (if any) developmental expression in *M. stipitatus*. Cluster III contains 419 genes expressed continually in *M. xanthus,* but not in *M. stipitatus*. Cluster IV consists of 408 genes expressed vegetatively in both species that show greater down-regulation in *M. xanthus*. Cluster V consists of 343 genes expressed developmentally in *M. stipitatus* after fruiting induction, but that are not consistently or highly upregulated in *M. xanthus*. Finally, Cluster VI consists of 342 genes that are expressed after fruiting induction in both species, although more strongly in the Δ*fruA* mutant of *M. xanthus*.

Interestingly, although we included the Δ*fruA* mutant in this study with the goal of separating fruiting specific genes from the general starvation response, the unbiased approach described above did not identify a gene cluster that is developmentally upregulated in both species and only upregulated in the presence of intact FruA. When we look at the genes in *M. xanthus* K-means cluster II (Fig. 5a), which are developmentally expressed genes whose expression is lower in the Δ*fruA* mutant, 115 out of 240 genes do not have matches in the *M. stipitatus* genome. Of the genes present in both genomes, however, the developmental transcriptional expression pattern is reproduced in approximately 105 of the genes present in *M. stipitatus* (Additional file 8) providing some confirmation of the shared role of FruA in the two species.

The COG enrichment patterns identified for each species individually are also visible in the orthologs. The vegetative genes (clusters I and IV) show increased representation of energy production (COG annotation C), translation related (COG annotation J) and Inorganic ion transport and metabolism (P). In contrast, the developmental clusters II and VI show substantial increases in genes putatively involved in signal transduction mechanisms (COG annotation T) and many of these genes seem to be inhibited by FruA, as they show higher expression in the Δ*fruA* strain. Cluster VI is of particular note as this cluster contains genes with substantial developmental upregulation in both species despite the acknowledged differences in the method used to induce fruiting body formation.

**Fig. 9:**
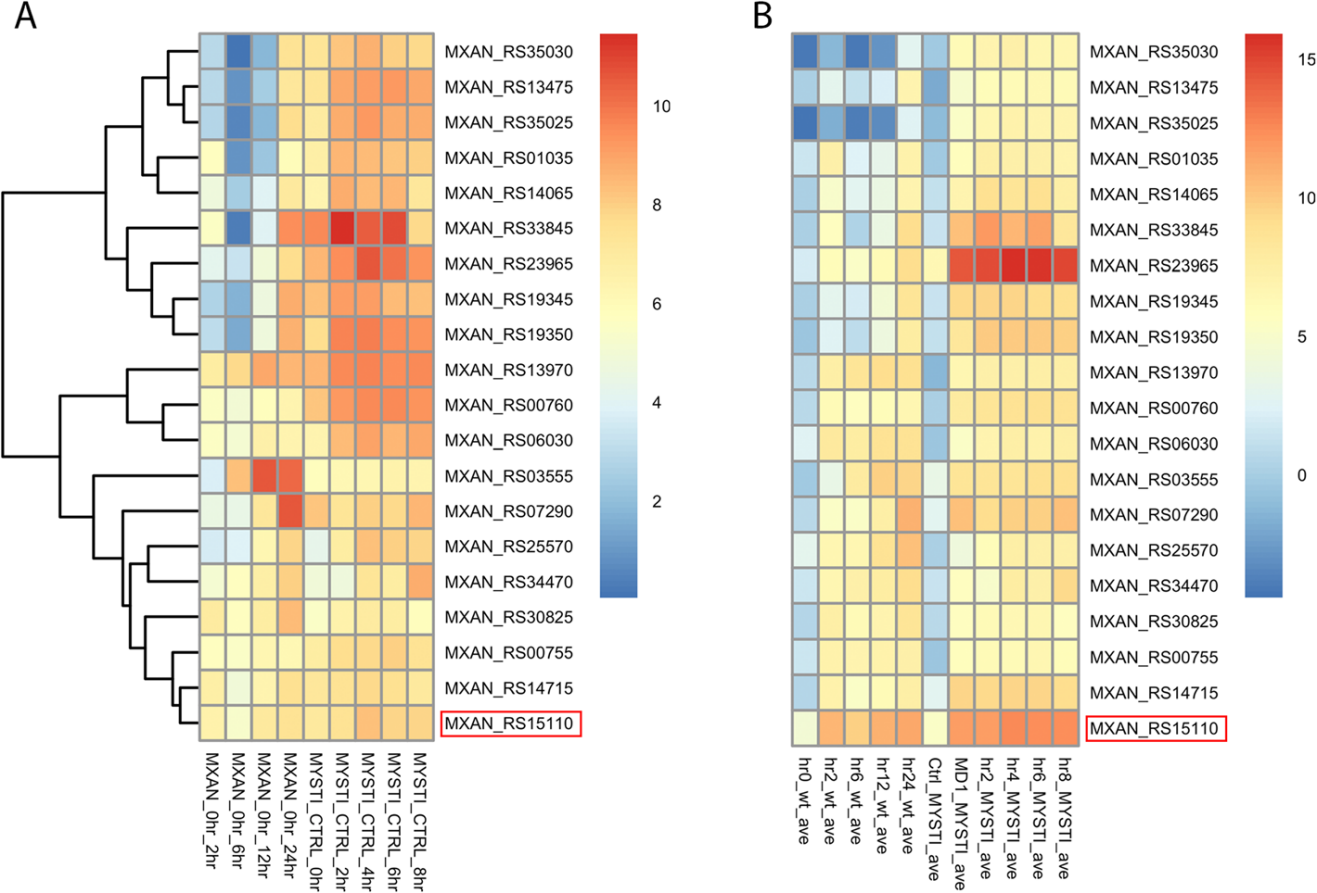
Heatmap clusters identify genes with similar expression patterns to a gene of interest, in this case *fruA*. An R code was used to extract the gene clusters that include *fruA* from a Euclidian distance dendrogram generated by Pheatmap of log2 fold change data for *M. xanthus* and *M. stipitatus* and a heatmap was generated for this cluster (**A**). Heatmaps were created to show the RPKM values for this set of genes (**B**)

We are optimistic that expression data from *M. stipitatus* can be used in future work to refine our understanding of critical developmental pathways in these unusual bacteria. To help identify candidate fruiting genes, we have developed a user-friendly R-script and are providing .csv files that will let users select a gene of interest and pull out clusters of genes with the most well-matched expression patterns (Additional files 33, 34, 35, 36 and 37). The script could also be used with RPKM data limited to specific clusters identified in the K-means analyses. We have tested this script by identifying the 19-gene cluster of genes whose log2fold-changes between vegetative and developmental timepoints for both *M. xanthus* and *M. stipitatus* most closely matched those of *fruA*. We then generated heatmaps visualizing both the changes in gene expression and the average RPKMs for the genes in this cluster (Fig. 9). Each of the genes in this cluster shows statistically significant changes in gene expression in at least one timepoint in the DEseq comparisons between WT and *ΔfruA* strains. This further suggests that these genes are directly or indirectly regulated by FruA, and demonstrates that our data can be mined to identify new candidate developmental genes.

## Conclusion

Here, we generated transcriptomes from multiple developmental timepoints from two species of fruiting myxobacteria, and from one non-developing mutant strain of *M. xanthus*. Recently, Muñoz-Dorado et al. described RNAseq-based developmental transcriptomes for *M. xanthus* [34]. This published work differs from ours as they continued the analysis significantly later in development (to 96 h) and did not include the early 2 h timepoint, since we focused specifically on the early commitment to fruiting, up through aggregation, and did not focus on sporulation. Also, they induced fruiting using a lower nutrient agar plate called CF medium instead of medium replacement of submerged cultures; while these techniques are often used interchangeably, sometimes specific genes are more involved in fruiting bodies formed via one or the other induction method. Finally, on a technical level, the authors did not deplete rRNA prior to sequencing. In contrast to our work, this means that Muñoz-Dorado et al. found that only 20% of genes had over 50 reads and sufficiently high concordance between replicates. This explains why we see much more of the genome showing statistically significant changes in regulation during development, and why we used the quantitative statistical tools DEseq and HTseq as the main basis for our analyses.

In contrast, the methods used in this work to induce fruiting body formation and to generate the transcriptomes are similar to those used by Sharma at al, so we were able to compare our results to theirs [35]. We carried out pairwise comparisons of data from all timepoints in both studies for both WT and *ΔfruA* strains, and looked at Pearson correlations, Spearman correlations, and scatter plots comparing expression for each gene (Additional file 9). Interestingly, we see notably higher levels of correlation using the Spearman Correlation rather than the Pearson correlation, which is consistent with the non-linear trends visible in the scatter plots. Notably, the best matches are between the vegetative samples, but our data best match later timepoints from the Sharma et al. results, where our 12 h sample best matches their 24 h sample, and our 24 h sample best matches their 72 h sample. Nonetheless, the data are well matched, suggesting that both independent studies properly capture these complex biological data.

Specifically, our data do corroborate many findings from Muñoz-Dorado et al., and Sharma et al. but also expand upon their data [34, 35]. We have demonstrated that large numbers of genes are regulated during fruiting body formation, although many of these gene expression changes still occur in a *ΔfruA* mutant in which fruiting bodies fail to form, and indeed FruA seems to be needed to reduce expression of many developmentally activated genes. Our data support that signal transduction genes and polyketide synthase genes are developmentally upregulated in *M. xanthus*, and as a gene-ontology category, signal transduction genes are also developmentally up-regulated in *M. stipitatus*, even if Interpro domain enrichment is less consistent in *M. stipitatus*. Additionally, metabolism and translation appear to be down-regulated during development in both species, although clearly these are broad categories and some genes assigned to each functional category are developmentally expressed. Also, despite substantial differences in the method of fruiting body induction, we were also able to identify clusters of genes whose expression patterns are similar between the two species, and were able to identify a set of genes with FruA dependent developmental upregulation that are conserved and have conserved expression in the two species. As several critical developmental genes are conserved and concordant in expression between the species, these data can support existing *M. xanthus* research projects by allowing researchers to examine the conservation of developmental processes in a second *Myxococcus* species, and can identify genes for future studies into the developmental role of previously uncharacterized genes and gene clusters. However, while there are genetic mechanisms shared between the two species, these data demonstrate that many developmental processes are unique to specific *Myxococcus* species, beginning with our inability to induce *M. stipitatus* fruiting when using the methodology most commonly used for *M. xanthus*. These practical and transcriptional differences highlight the need for more comparative work in the future.

## Methods

### Bacterial strains and media

All work was carried out using *M. xanthus* strain DK1622 (NC_008095.1), or *M. stipitatus* strain DSM14675 (NC_020126.1) [44, 48, 63, 64]. *M. xanthus* was routinely cultivated in CTT (1% casitone, 10 mM Tris-HCl pH 7.6, 8 mM MgSO_4_, 1 mM potassium phosphate, pH 7.6) or CTT with 1.5% agar [65]. *M. stipitatus* was routinely cultivated in a modified MD1 (0.3% Casitone, 5 mM CaCl_2_, 8.1 mM MgSO_4_, 0.5 mg vitamin B_12_, 0.15% EDTA, 1 μM ZnCl_2_, 1 μm CuSO_4_, 1 μM CoCl_2_, 1 μm Na_2_Mo_4_, 1 μm MnSO_4_, and 10 μm FeSO_4_) or in “MD1 + CAS” (modified MD1 containing 1% casitone) and on plates of the same media including 1.5% agar. *M. stipitatus* fruiting body formation was induced on starvation plates (0.1% CaCl_2_ and 1.5% agar) [66, 67].

### Induction of development

*M. xanthus* development was carried out in submerged culture as described previously [68]; briefly, 150 μl of cells from an overnight culture, concentrated to an OD_550_ of 7.0, were inoculated into 16 ml CTT, allowed to settle on the bottom of 85 mm diameter polystyrene petri dishes, and after 24 h incubation at 32 degrees, the CTT was carefully replaced with MC7 (100 mM MOPS pH 7 and 10 mM CaCl_2_) and plates were incubated at 32 degrees. Cells were harvested at 2, 6, 12 and 24 h. For *M. stipitatus*, cells growing on MD1 plates were inoculated into 2 ml MD1 medium, incubated in an orbital shaking incubator overnight at 32 degrees, and the next day scaled up to 10 ml cultures. These cultures again incubated overnight, and were then split into 20 ml MD1 + CAS or 85 ml MD1 and incubated overnight at 32 degrees. Aliquots of the MD1 + CAS and MD1 cultures were harvested directly for RNA as control samples, and the remainder of the MD1 culture was collected, concentrated, washed with MC7, resuspended to a calculated OD of 7.0, and 700 μl was spread onto starvation plates, and dried for 15 min. in a laminar flow hood. Plates were then incubated at 32 degrees, and cells were harvested at 2, 4, 6 and 8 h.

### Isolation of RNA

RNA was isolated from harvested cells by hot-phenol extraction as previously described [69]. Isolated nucleic acids were then treated for 2 h with DNase1 to digest DNA, and DNase was removed by phenol-chloroform extraction [53].

### RNAseq

RNA samples were quality tested by bioanalyzer and libraries were prepared following standard protocols and Illumina reagents, including Illumina Ribo-Zero rRNA depletion [70], by the Max Planck Genome Center, Cologne, Germany, and were sequenced with single-end 100 bp reads on the Illumina HiSeq2500.

### Sequence analyses and bioinformatics analyses

RNAseq reads were trimmed to remove adapter sequences and poor quality bases using Trimmomatic, aligned to the *M. xanthus* DK1622 genome using BWA MEM, reads were mapped to specific genes and quantified using HTseq, and read counts were compared between timepoints or strains using DEseq [55, 56, 71–73]. Because we opted to rRNA deplete our samples during library production, we manually removed the rRNA genes from the gene annotation files used for read counting by HTseq and DESeq. Comparisons between WT and *ΔfruA* and between *M. xanthus* and *M. stipitatus* were made using Microsoft Excel and R scripts. Gene orthologies and synteny between *M. xanthus* and *M. stipitatus* were assigned using EDGAR 2.0 [51, 52]. Graphs and figures were generated using R scripts including BioVenn, HTseq, apeglm, pheatmap, and Enhanced Volcano [55, 74–77]. Interpro and gene ontology enrichments were calculated using the web application iDEP using standard settings [59, 60]. This combined DEseq analysis with enrichment analysis using GAGE and ontological information from ENSEMBL and stringdb to calculate enrichment for time point comparisons [55, 61, 78].

## Supplementary Information

**Additional file 1: Supplemental figure 1.** Known fruiting body related genes in *M. xanthus* show varied patterns of gene expression when present in *M. stipitatus*. The R package pheatmap was used to create a heatmap of the average log_2_ RPKMs for the 95 previously described fruiting body related genes. If the indicated gene is not present in *M. stipitatus*, the boxes corresponding to expression in this species are grey. The gene designations in this figure are from the original genome annotation and the names are those used in publications.

**Additional file 2 Supplemental Fig. 2.** Many genes show statistically significant developmental regulation during *M. xanthus* fruiting body formation. The data presented in Fig. 2 are presented here a Volcano plots for each pairwise comparison. Volcano plots were generated using the “Enhanced volcano” R package, with points highlighted in red having Log2 fold changes of ≤ − 2 or ≥ 2, and *p*-values of ≤1 × 10^− 3^.

**Additional file 3: Supplemental table 1.** Interpro domains that are enriched in each of the 4 K-means clusters for *M. xanthus.*

**Additional file 4: Supplemental figure 3.** Interpro enrichments identify protein families that are developmentally regulated in *M. xanthus* and *M. stipitatus*. Enrichments of interpro were identified for pairwise comparisons of developmental timepoints to HR0 of WT *M. xanthus* and to the matched timepoint of the Δ*fruA* strain (A) and pairwise comparisons of *M. stiptatus* developmental timepoints compared to the vegetative control (B). Upregulation in the developmental timepoints is shown in purple, downregulation is shown in green according to the GAGE enrichment statistics.

**Additional file 5: Supplemental figure 4.** Enrichment of protein families using pfam annotations supports enrichments identified with interpro categories. Analysis was carried out as for Fig. 5, except pfam protein family annotations were used. Enrichments were identified for pairwise comparisons of developmental timepoints to HR0 of WT *M. xanthus* and to the matched timepoint of the Δ*fruA* strain (A) and pairwise comparisons of *M. stiptatus* developmental timepoints compared to the vegetative control (B). Upregulation in the developmental timepoints is shown in purple, downregulation is shown in green according to the GAGE enrichment statistics.

**Additional file 6: Supplemental figure 5.** FruA protein is present in *M. stipitatus* cells grown in MD1, but not in Rich medium. *M. stipitatus* cultures grown in media with varied casitone concentrations (0.3, 0.6, and 1%), including samples of the specific cultures used for this transcriptomic work, were lysed and subjected to Western Blot analysis for the FruA protein.

**Additional file 7: Supplemental figure 6.** Core genes shared by *M. xanthus* and *M. stipitatus* show correlated expression levels under vegetative conditions but less correlation during development. For each sample, read counts generated by HTseq were converted to fragments per million reads, and were averaged over the biological replicates from each strain and timepoint or condition. Then all timepoints or conditions were compared via scatter plots between the two species in a pairwise fashion.

**Additional file 8: Supplemental figure 7.** Heatmap of log2 RPKM expression levels for conserved genes in *M. xanthus* K-means cluster II for all 3 strains demonstrates that conserved genes within this cluster are also developmentally up-regulated in *M. stipitatus*.

**Additional file 9: Supplemental figure 8.** Comparisons between this *M. xanthus* transcriptome with that generated by Sharma et al., 2020 shows high levels of correlation. A. Pearson correlation of data for each timepoint comparing results from Sharma et al. on the X axis and those generated in this work on the Y-axis. R values range from 0.15–0.4 (Blue to Orange). B. Spearman correlations comparing the Sharma et al. transcriptomes on the X axis to those presented in this work on the Y axis. R values range from 0.6–0.9. C - Scatterplot of log2 values for WT expression from the best matched timepoints for each transcriptome, with the data generated in this work on the Y-axis and from Sharma et al. on the X-axis. D - Scatterplot of log2 values for the same timepoint comparisons in panel C except showing Δ*fruA* mutant expression on the Y-axis and Sharma et al. on the X-axis.

**Additional files 10: **Uncropped image of *M. xanthus* 0 h culture.

**Additional files 11: **Uncropped image of *M. xanthus* 2 h culture.

**Additional files 12: **Uncropped image of *M. xanthus* 6 h culture.

**Additional files 13: **Uncropped image of *M. xanthus* 12 h culture.

**Additional files 14: **Uncropped image of *M. xanthus* 24 h culture.

**Additional files 15: **Uncropped image of *M. xanthus* 54 h culture.

**Additional files 16: **Uncropped image of *M. stipitatus* 2 h culture.

**Additional files 17: **Uncropped image of *M. stipitatus* 4 h culture.

**Additional files 18: **Uncropped image of *M. stipitatus* 7 h culture.

**Additional files 19: **Uncropped image of *M. stipitatus* 26 h culture.

**Additional files 20: **Uncropped image of *M. stipitatus* fruiting body side view.

**Additional file 21:.** Western blot scan, 2 min exposure

**Additional file 22:.** Western blot scan, 30 s exposure

**Additional file 23:.** New and old locus name correspondences

**Additional file 24: **WT *M. xanthus* read counts and DEseq data

**Additional file 25: **DEseq data for timepoint comparisons between *ΔfruA* and WT *M. xanthus*

**Additional file 26: ***ΔfruA M. xanthus* read counts and DEseq data

**Additional file 27: **WT *M. stipitatus* read counts and DEseq data

**Additional file 28:.** Percentages of total annotations in each COG category in each Kmeans cluster

**Additional file 29: **Kmeans clusters for *M. xanthus.*

**Additional file 30: **Kmeans clusters for *M. stipitatus*

**Additional file 31:.** Kmeans clusters for top orthologs

**Additional file 32: **Log_2_ Fold-changes for *M. xanthus*, *M. stipitatus* core genes

**Additional file 33: **RPKM for all *M. xanthus*, *M. stipitatus* core genes

**Additional file 34: **RPKM for all WT and Δ*fruA M. xanthus*.

**Additional file 35: **RPKM log_2_ for all *M. xanthus*, *M. stipitatus* core genes

**Additional file 36: **RPKM log_2_ for all WT and Δ*fruA M. xanthus*

**Additional file 37:.** R code for identifying gene clusters containing target gene

## Data Availability

All read counts and DEseq data are available as Additional files S7-S10. The sequence data has been deposited in the NCBI SRA database. *M. xanthus* sequences can be accessed in BioProject PRJNA705214 http://www.ncbi.nlm.nih.gov/bioproject/705214. *M. stipitatus* sequences can be accessed in BioProject PRJNA705220 http://www.ncbi.nlm.nih.gov/bioproject/705220.
