# Supplementary figures and images for: Transcriptomic analysis of the Myxococcus xanthus FruA regulon, and comparative developmental transcriptomic analysis of two fruiting body forming species, Myxococcus xanthus and Myxococcus stipitatus
Source: BMC Genomics. 2021 Nov 1;22:784. doi: 10.1186/s12864-021-08051-w (PMC8561891; doi:10.1186/s12864-021-08051-w)

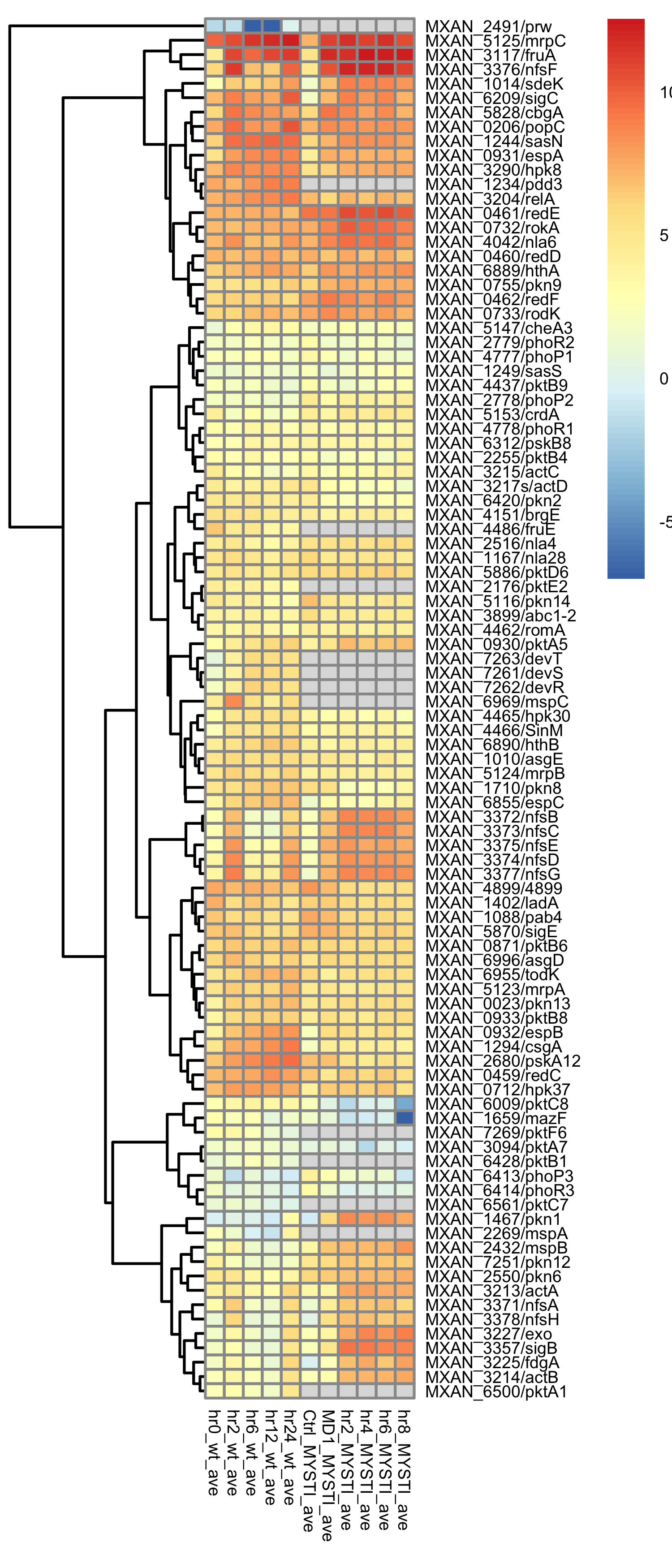

Supplement: Supplementary file 1 — Additional file 1: Supplemental figure 1. Known fruiting body related genes in M. xanthus show varied patterns of gene expression when present in M. stipitatus. The R package pheatmap was used to create a heatmap of the average log2 RPKMs for the 95 previously described fruiting body related genes. If the indicated gene is not present in M. stipitatus, the boxes corresponding to expression in this species are grey. The gene designations in this figure are from the original genome annotation and the names are those used in publications. [file 12864_2021_8051_MOESM1_ESM.jpg]

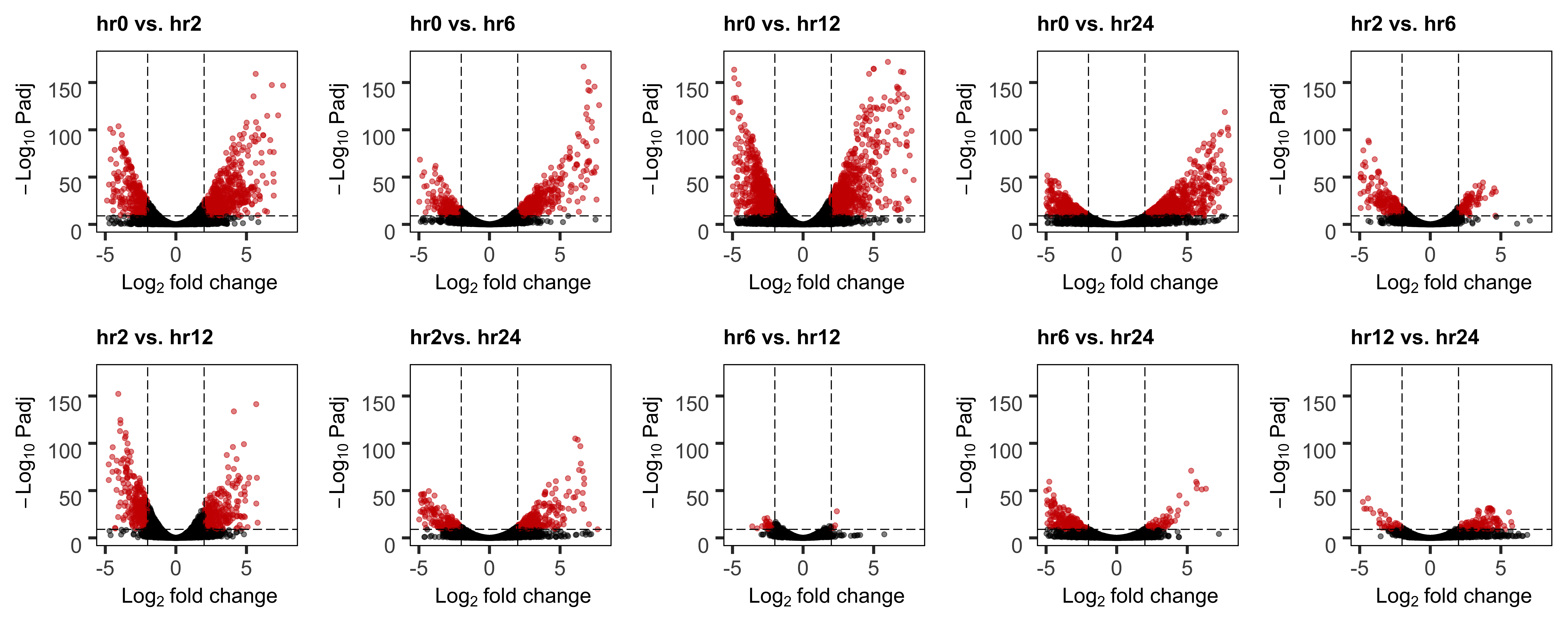

Supplement: Supplementary file 2 — Additional file 2 Supplemental Fig. 2. Many genes show statistically significant developmental regulation during M. xanthus fruiting body formation. The data presented in Fig. 2 are presented here a Volcano plots for each pairwise comparison. Volcano plots were generated using the “Enhanced volcano” R package, with points highlighted in red having Log2 fold changes of ≤ − 2 or ≥ 2, and p-values of ≤1 × 10− 3. [file 12864_2021_8051_MOESM2_ESM.jpg]

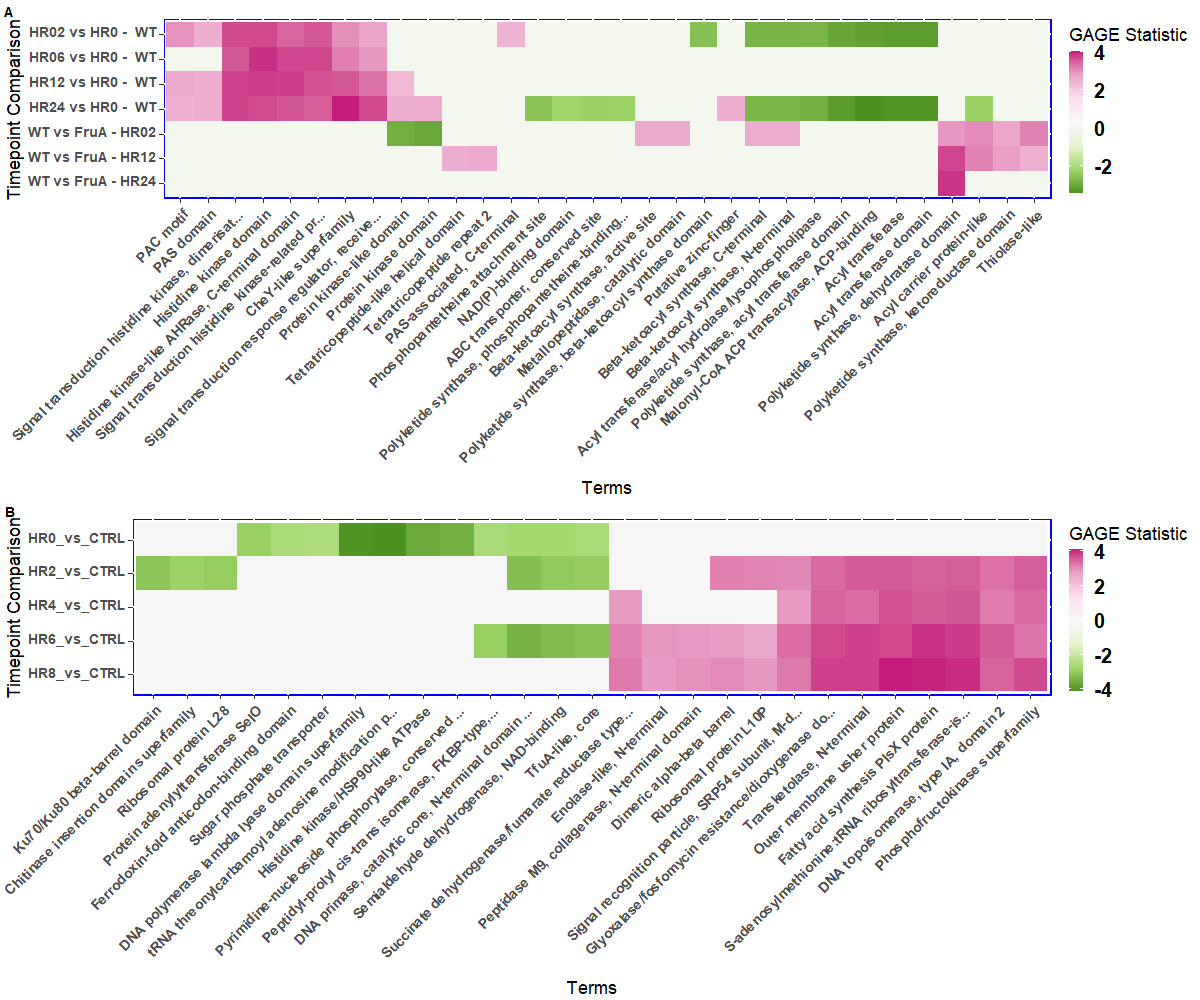

Supplement: Supplementary file 4 — Additional file 4: Supplemental figure 3. Interpro enrichments identify protein families that are developmentally regulated in M. xanthus and M. stipitatus. Enrichments of interpro were identified for pairwise comparisons of developmental timepoints to HR0 of WT M. xanthus and to the matched timepoint of the ΔfruA strain (A) and pairwise comparisons of M. stiptatus developmental timepoints compared to the vegetative control (B). Upregulation in the developmental timepoints is shown in purple, downregulation is shown in green according to the GAGE enrichment statistics. [file 12864_2021_8051_MOESM4_ESM.tiff]

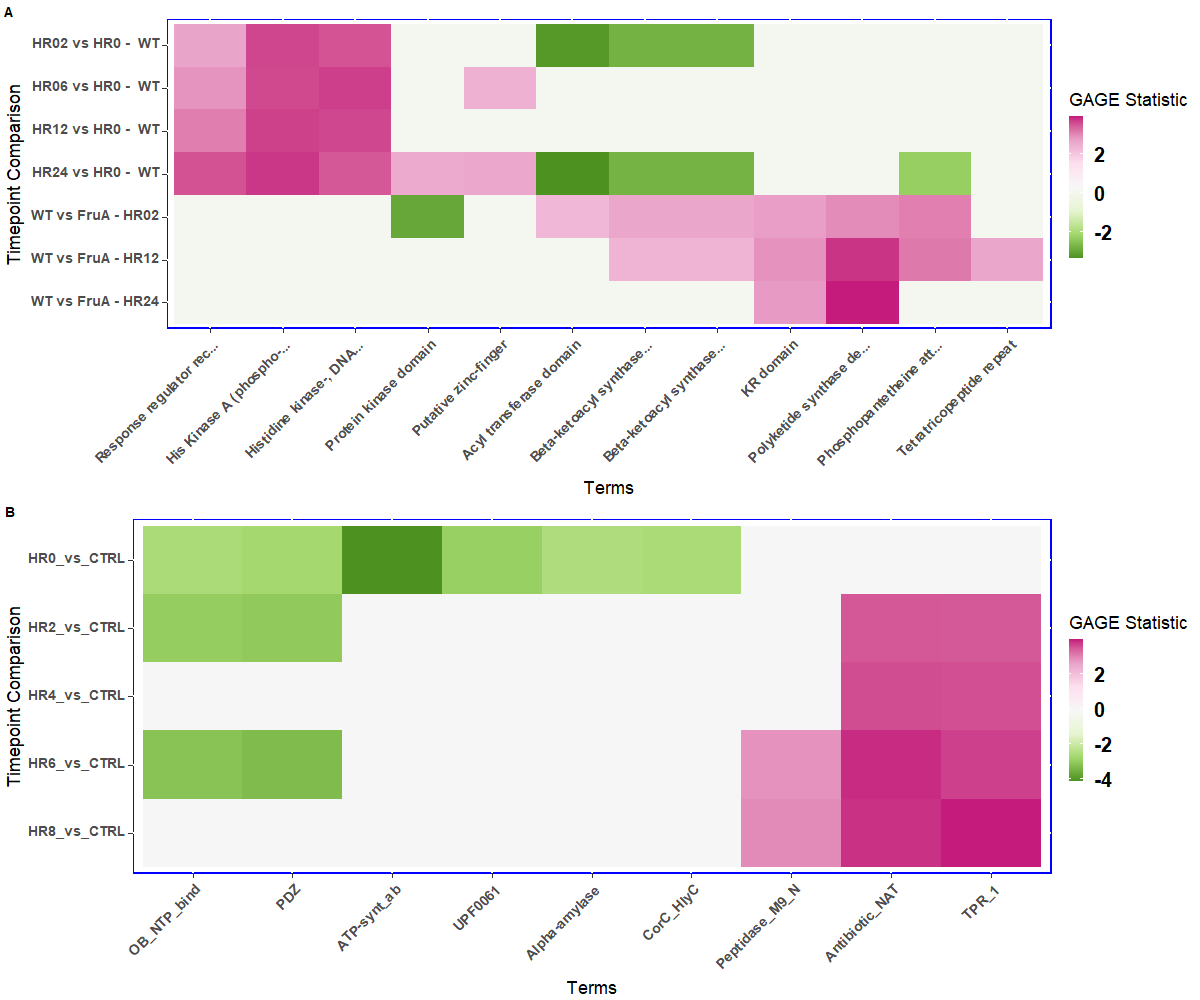

Supplement: Supplementary file 5 — Additional file 5: Supplemental figure 4. Enrichment of protein families using pfam annotations supports enrichments identified with interpro categories. Analysis was carried out as for Fig. 5, except pfam protein family annotations were used. Enrichments were identified for pairwise comparisons of developmental timepoints to HR0 of WT M. xanthus and to the matched timepoint of the ΔfruA strain (A) and pairwise comparisons of M. stiptatus developmental timepoints compared to the vegetative control (B). Upregulation in the developmental timepoints is shown in purple, downregulation is shown in green according to the GAGE enrichment statistics. [file 12864_2021_8051_MOESM5_ESM.tiff]

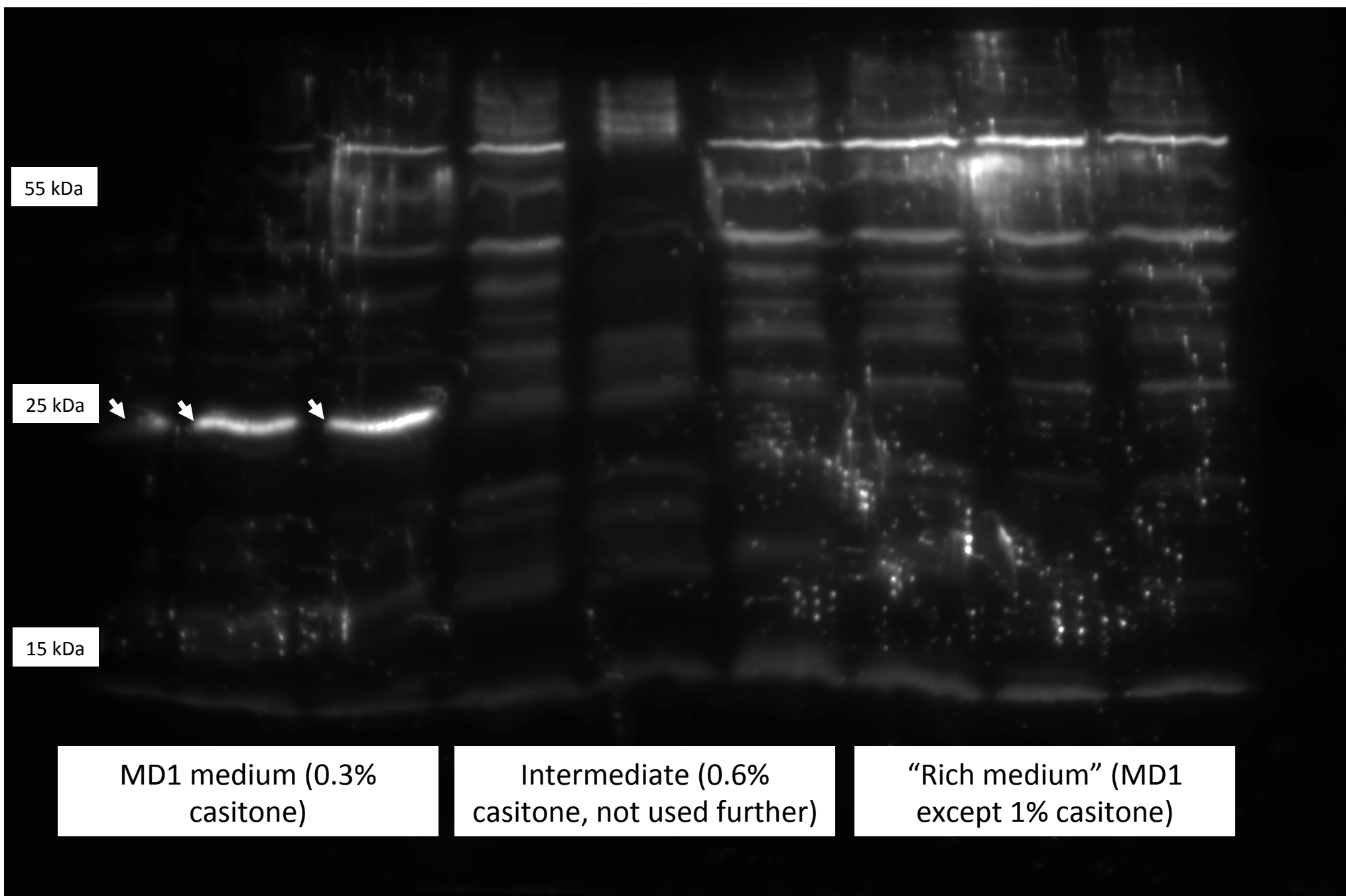

Supplement: Supplementary file 6 — Additional file 6: Supplemental figure 5. FruA protein is present in M. stipitatus cells grown in MD1, but not in Rich medium. M. stipitatus cultures grown in media with varied casitone concentrations (0.3, 0.6, and 1%), including samples of the specific cultures used for this transcriptomic work, were lysed and subjected to Western Blot analysis for the FruA protein. [file 12864_2021_8051_MOESM6_ESM.pdf]

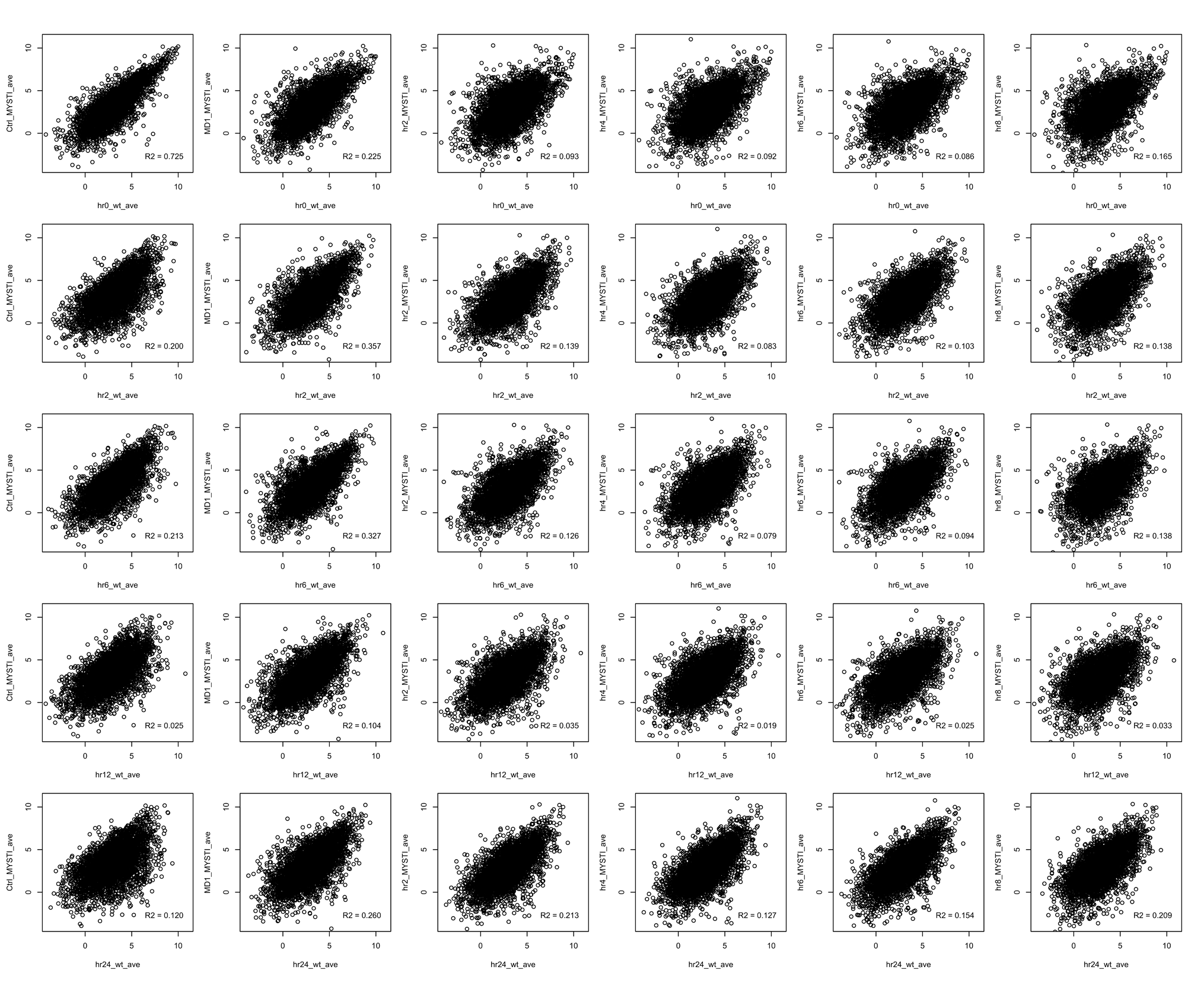

Supplement: Supplementary file 7 — Additional file 7: Supplemental figure 6. Core genes shared by M. xanthus and M. stipitatus show correlated expression levels under vegetative conditions but less correlation during development. For each sample, read counts generated by HTseq were converted to fragments per million reads, and were averaged over the biological replicates from each strain and timepoint or condition. Then all timepoints or conditions were compared via scatter plots between the two species in a pairwise fashion. [file 12864_2021_8051_MOESM7_ESM.tif]

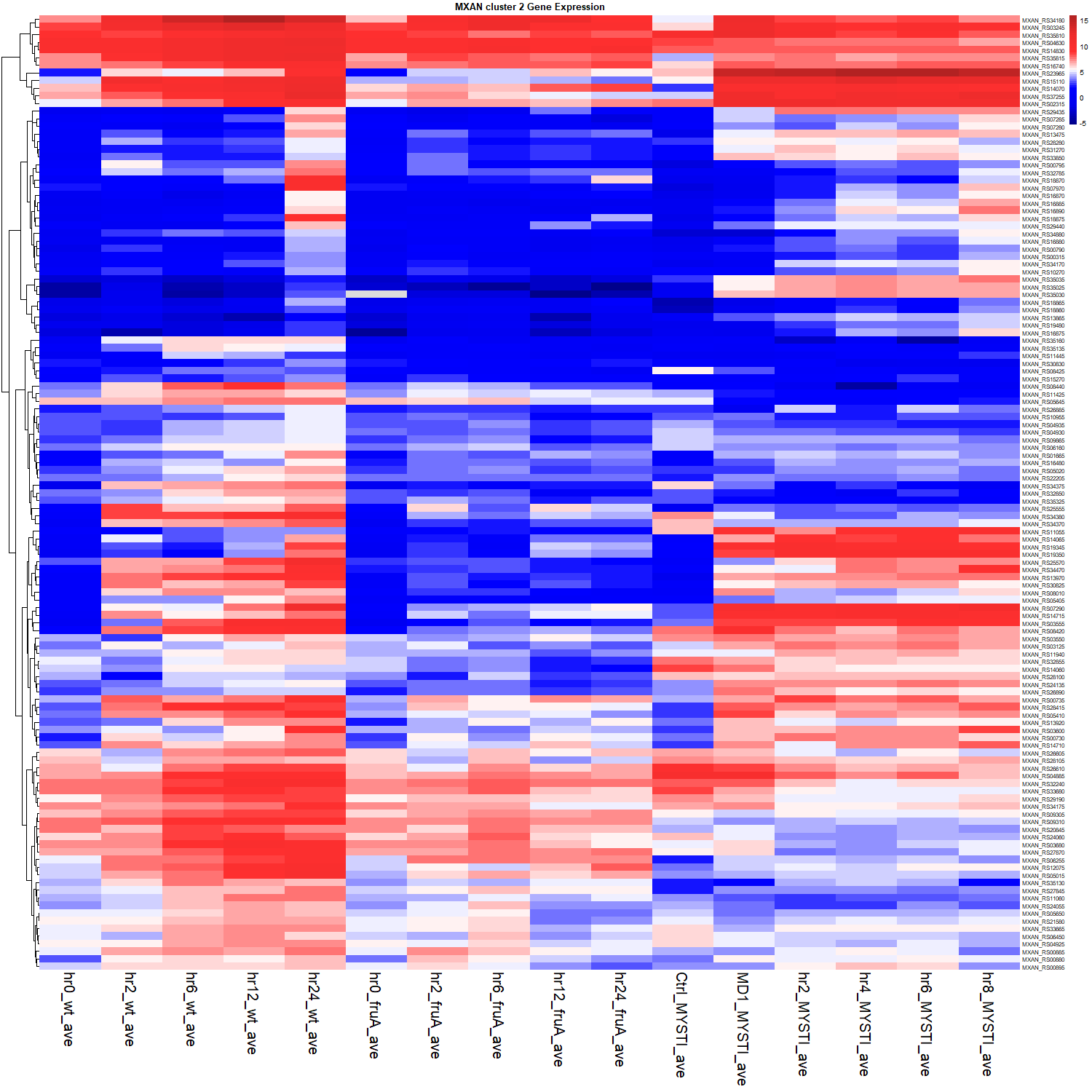

Supplement: Supplementary file 8 — Additional file 8: Supplemental figure 7. Heatmap of log2 RPKM expression levels for conserved genes in M. xanthus K-means cluster II for all 3 strains demonstrates that conserved genes within this cluster are also developmentally up-regulated in M. stipitatus. [file 12864_2021_8051_MOESM8_ESM.tiff]

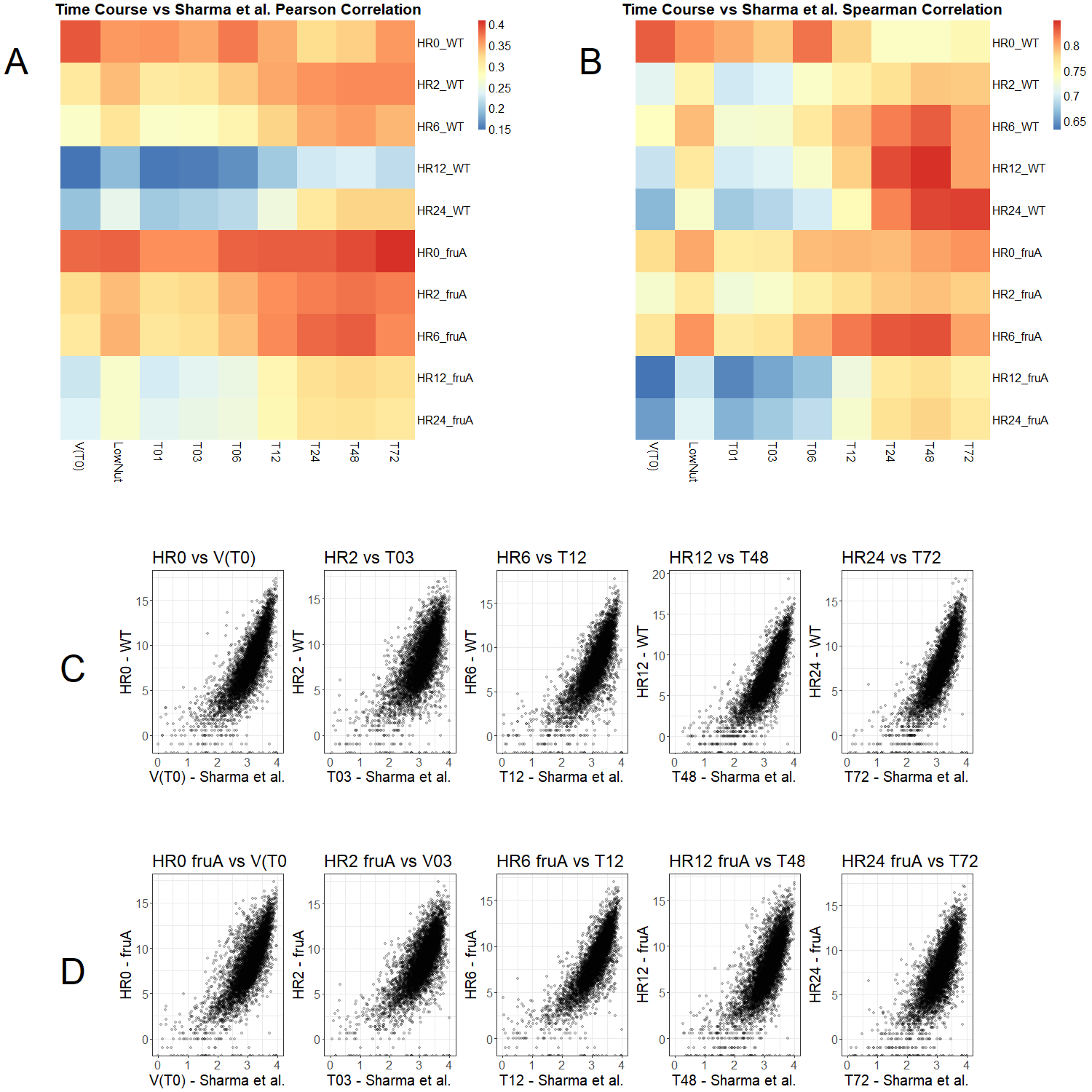

Supplement: Supplementary file 9 — Additional file 9: Supplemental figure 8. Comparisons between this M. xanthus transcriptome with that generated by Sharma et al., 2020 shows high levels of correlation. A. Pearson correlation of data for each timepoint comparing results from Sharma et al. on the X axis and those generated in this work on the Y-axis. R values range from 0.15–0.4 (Blue to Orange). B. Spearman correlations comparing the Sharma et al. transcriptomes on the X axis to those presented in this work on the Y axis. R values range from 0.6–0.9. C - Scatterplot of log2 values for WT expression from the best matched timepoints for each transcriptome, with the data generated in this work on the Y-axis and from Sharma et al. on the X-axis. D - Scatterplot of log2 values for the same timepoint comparisons in panel C except showing ΔfruA mutant expression on the Y-axis and Sharma et al. on the X-axis. [file 12864_2021_8051_MOESM9_ESM.tiff]

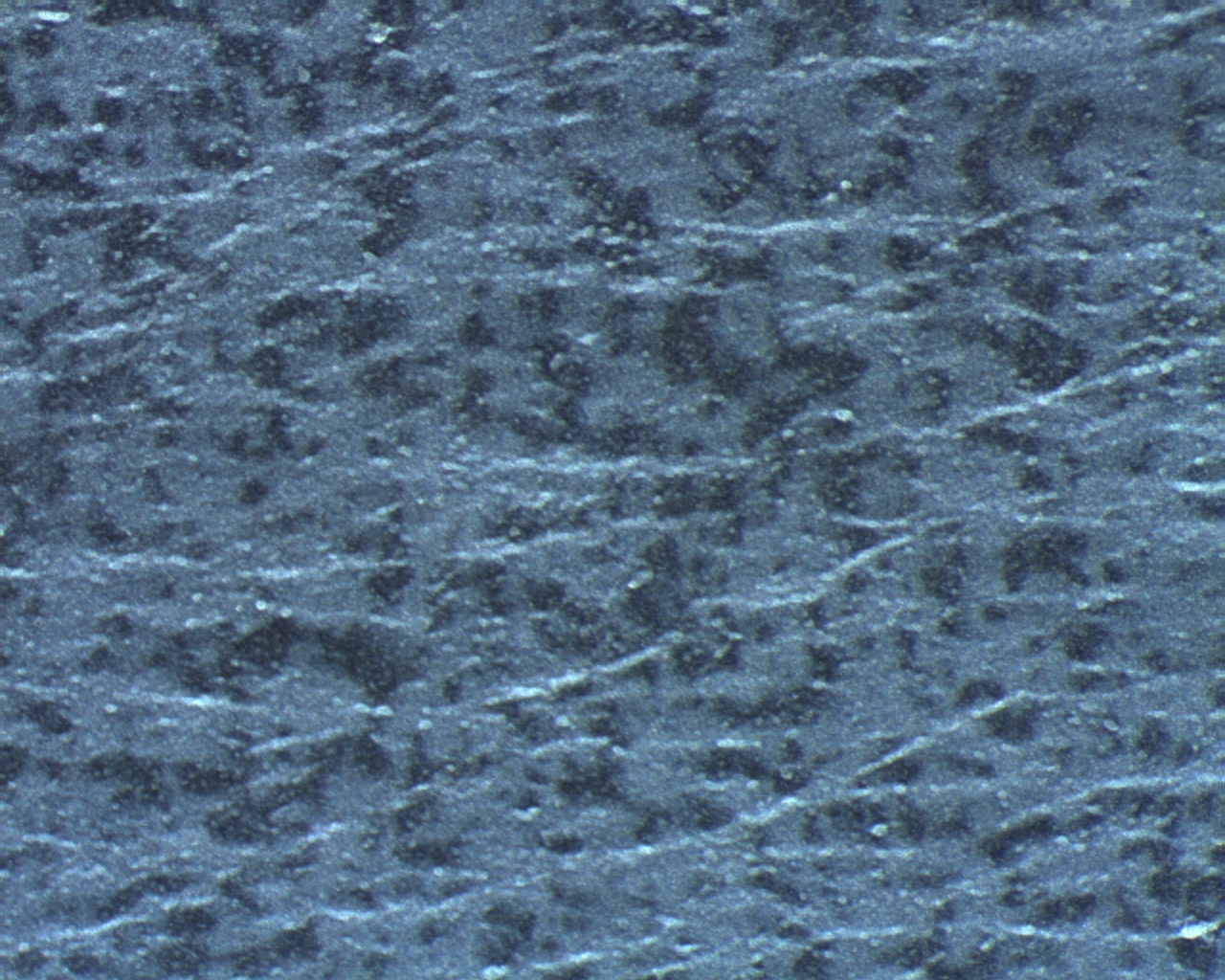

Supplement: Supplementary file 10 — Additional files 10: Uncropped image of M. xanthus 0 h culture. [file 12864_2021_8051_MOESM10_ESM.jpg]

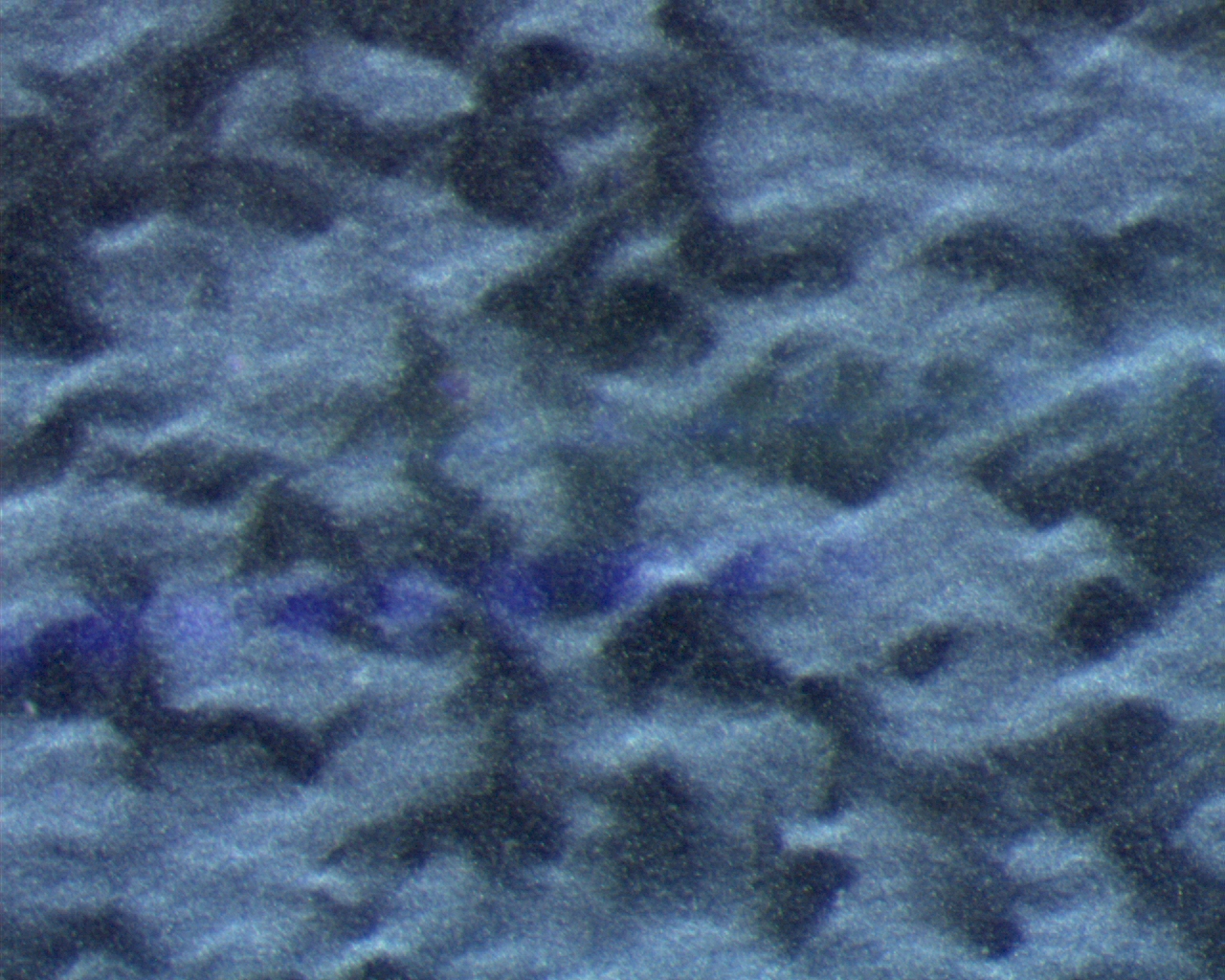

Supplement: Supplementary file 11 — Additional files 11: Uncropped image of M. xanthus 2 h culture. [file 12864_2021_8051_MOESM11_ESM.jpg]

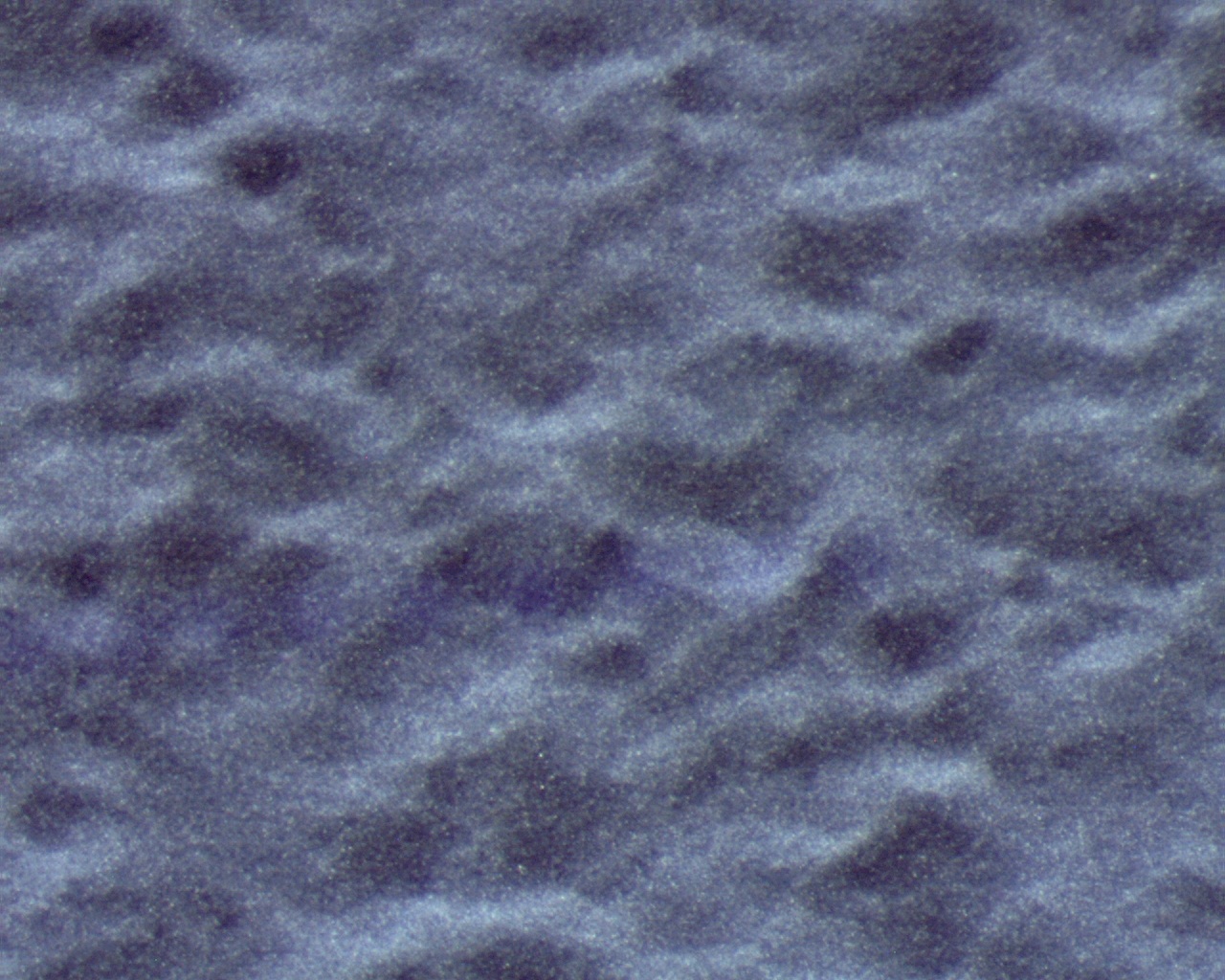

Supplement: Supplementary file 12 — Additional files 12: Uncropped image of M. xanthus 6 h culture. [file 12864_2021_8051_MOESM12_ESM.jpg]

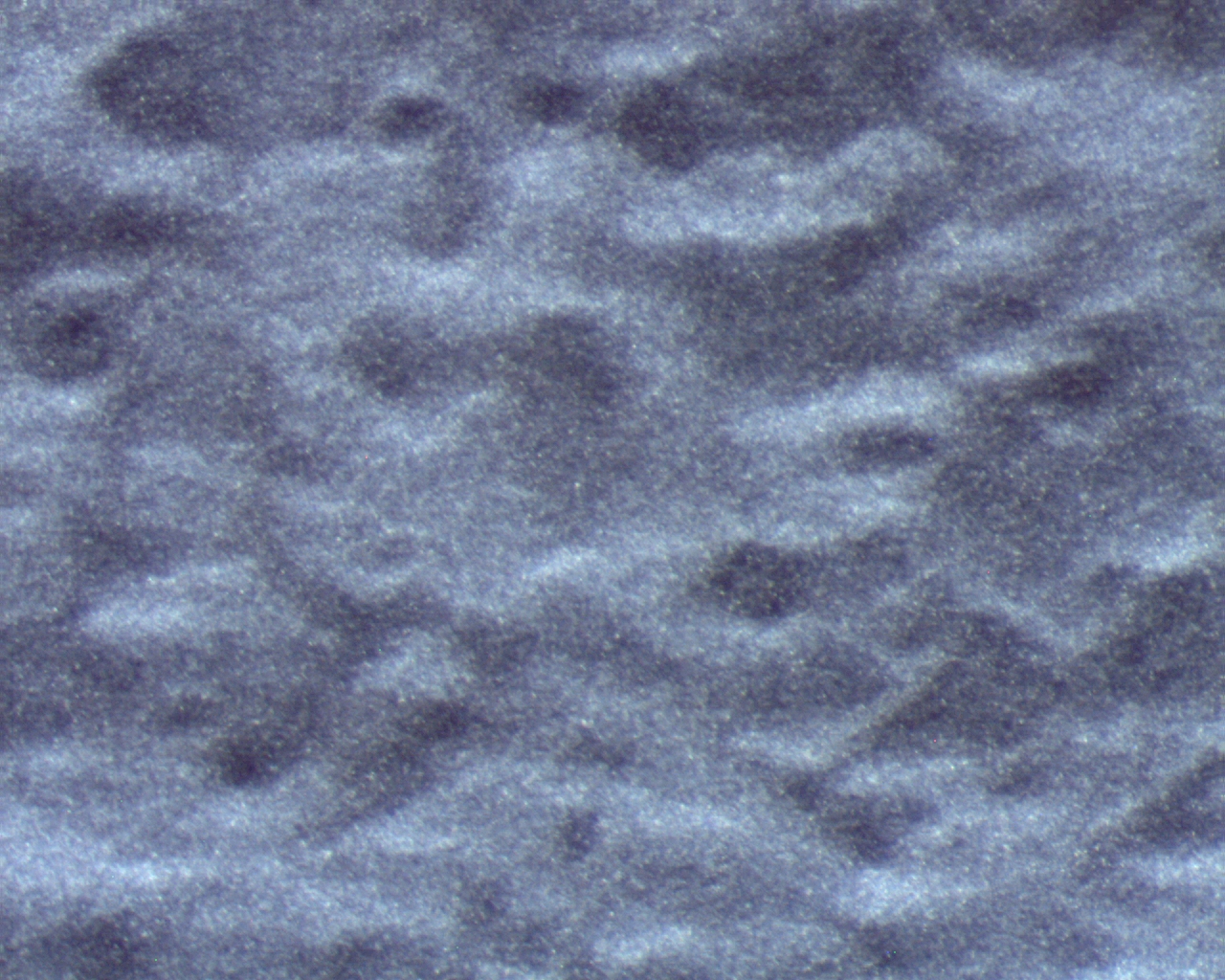

Supplement: Supplementary file 13 — Additional files 13: Uncropped image of M. xanthus 12 h culture. [file 12864_2021_8051_MOESM13_ESM.jpg]

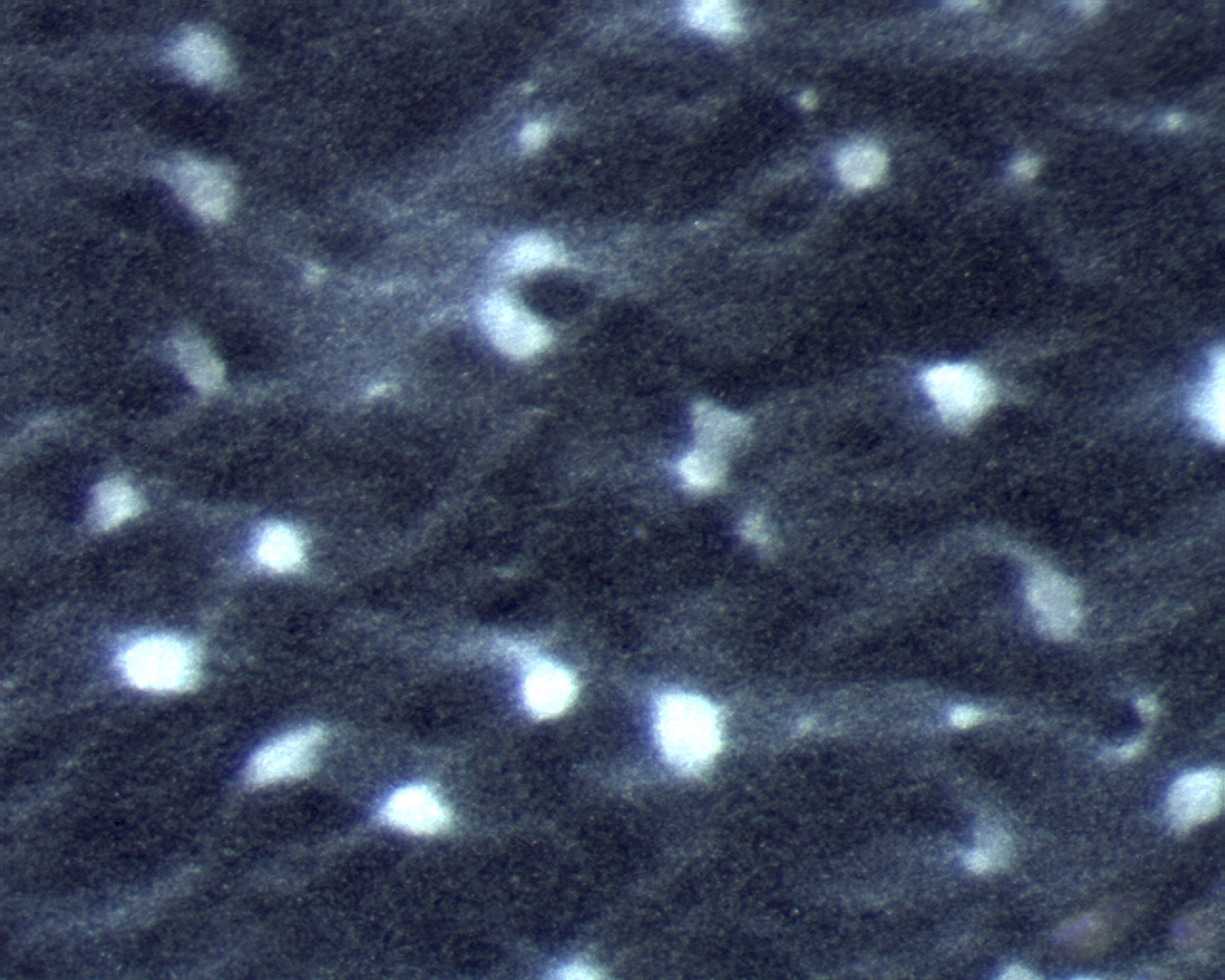

Supplement: Supplementary file 14 — Additional files 14: Uncropped image of M. xanthus 24 h culture. [file 12864_2021_8051_MOESM14_ESM.jpg]

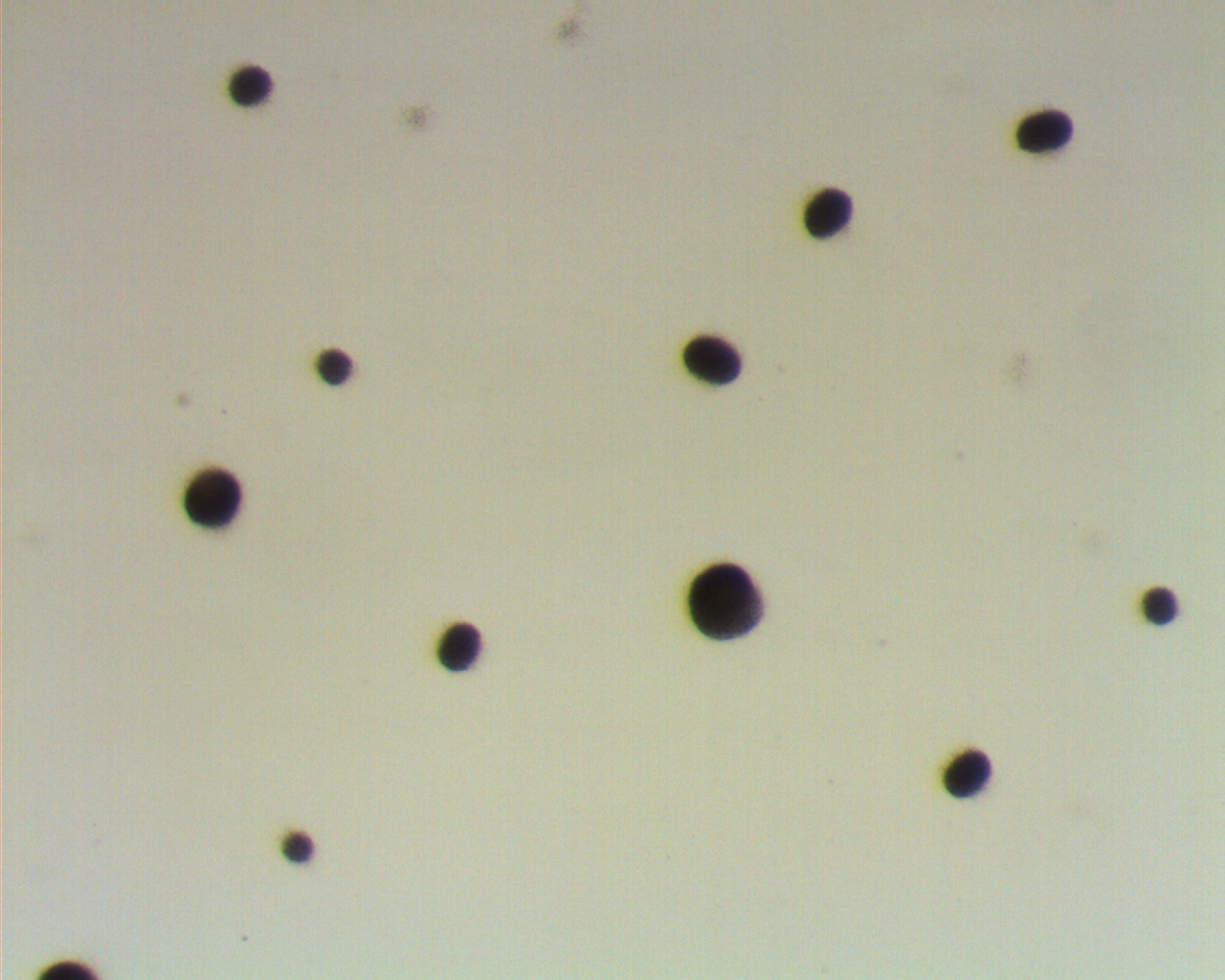

Supplement: Supplementary file 15 — Additional files 15: Uncropped image of M. xanthus 54 h culture. [file 12864_2021_8051_MOESM15_ESM.jpg]

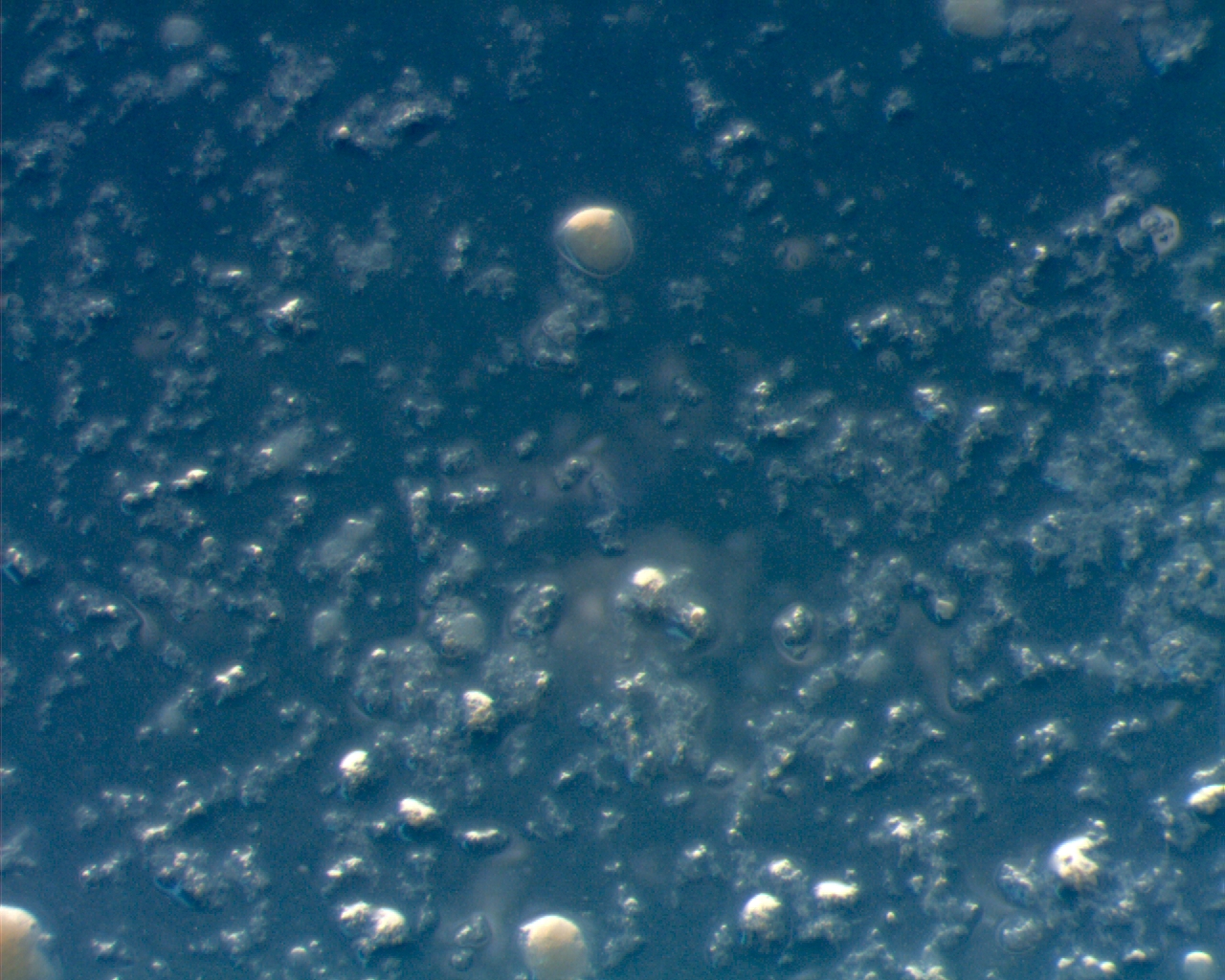

Supplement: Supplementary file 16 — Additional files 16: Uncropped image of M. stipitatus 2 h culture. [file 12864_2021_8051_MOESM16_ESM.jpg]

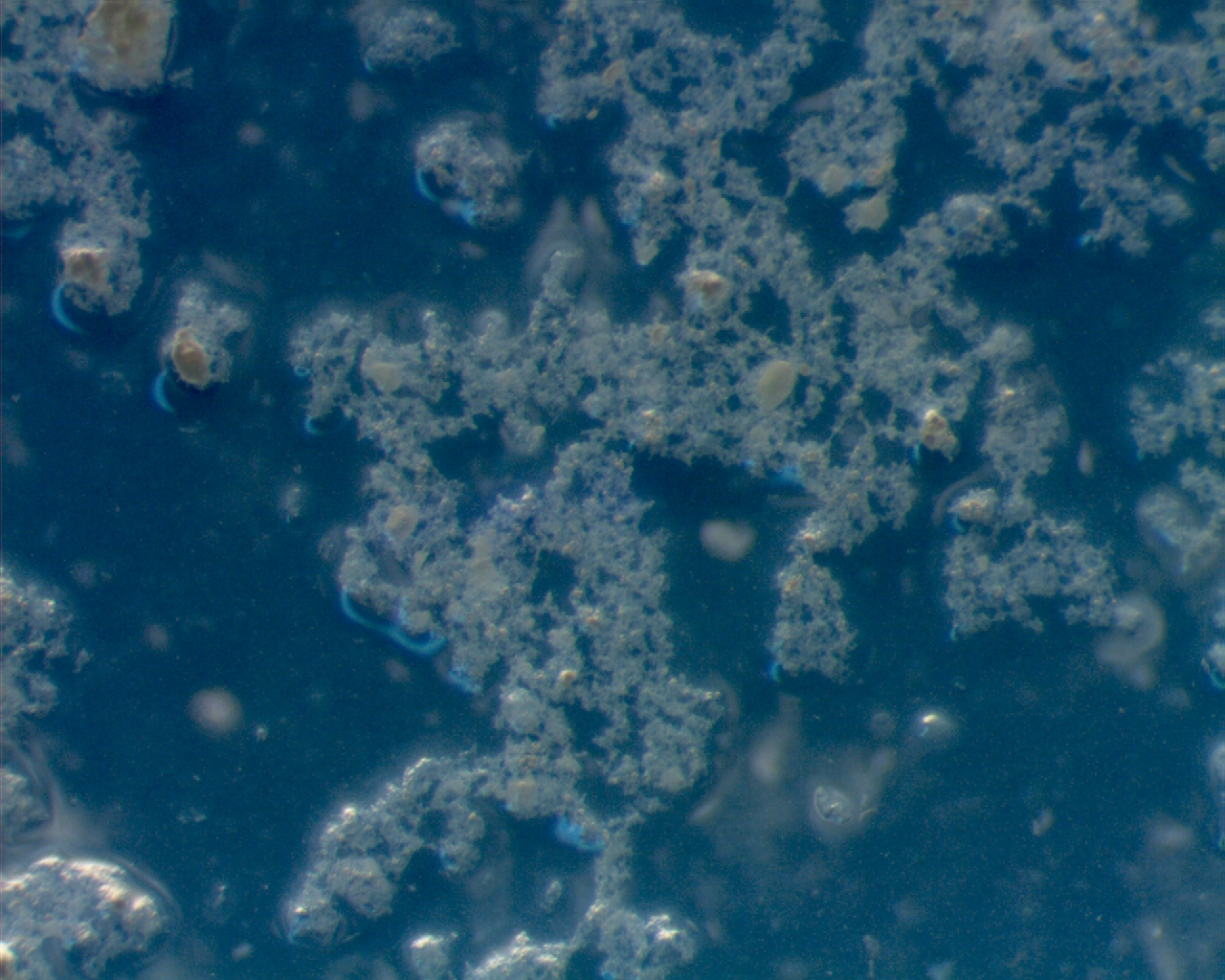

Supplement: Supplementary file 17 — Additional files 17: Uncropped image of M. stipitatus 4 h culture. [file 12864_2021_8051_MOESM17_ESM.jpg]

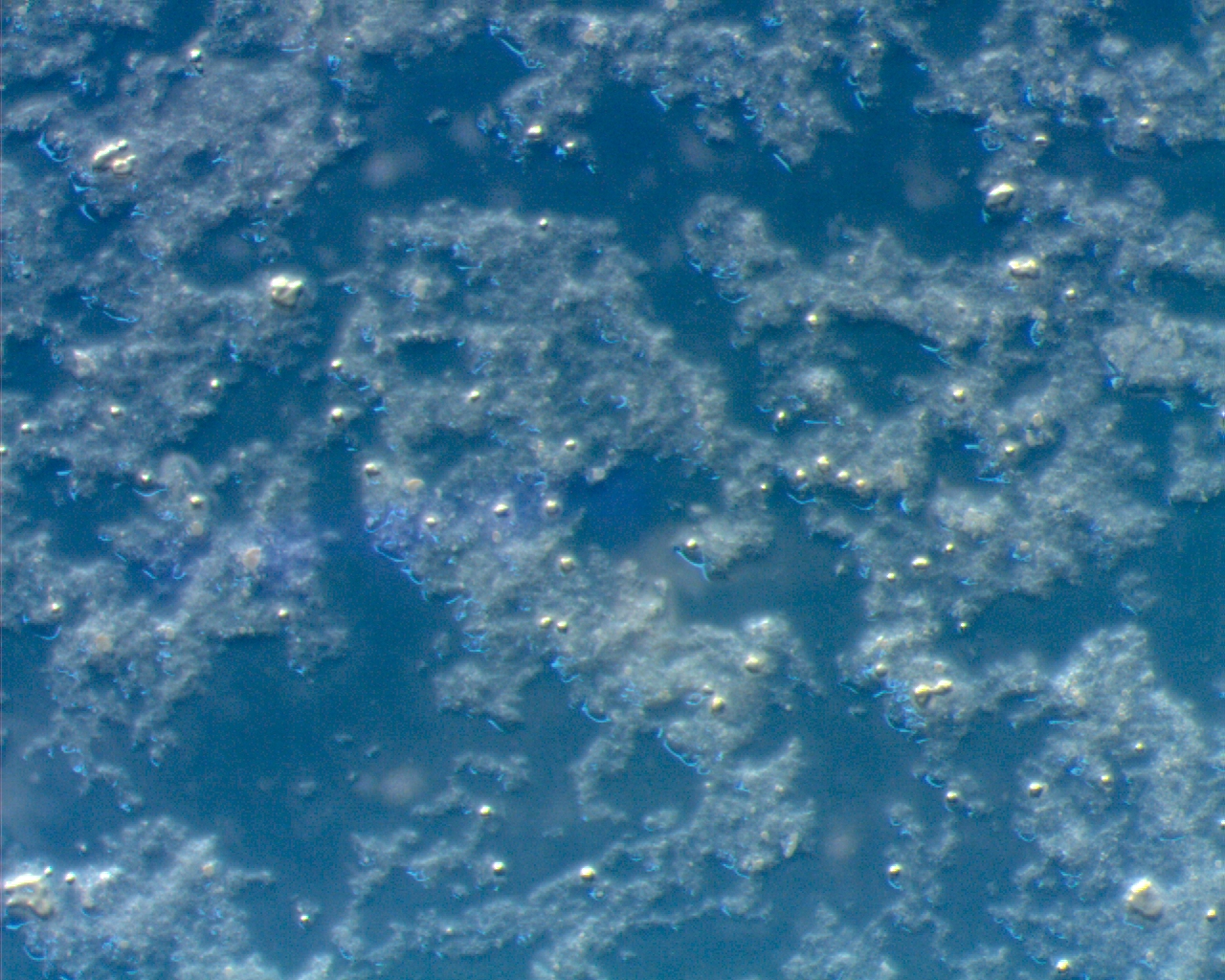

Supplement: Supplementary file 18 — Additional files 18: Uncropped image of M. stipitatus 7 h culture. [file 12864_2021_8051_MOESM18_ESM.jpg]

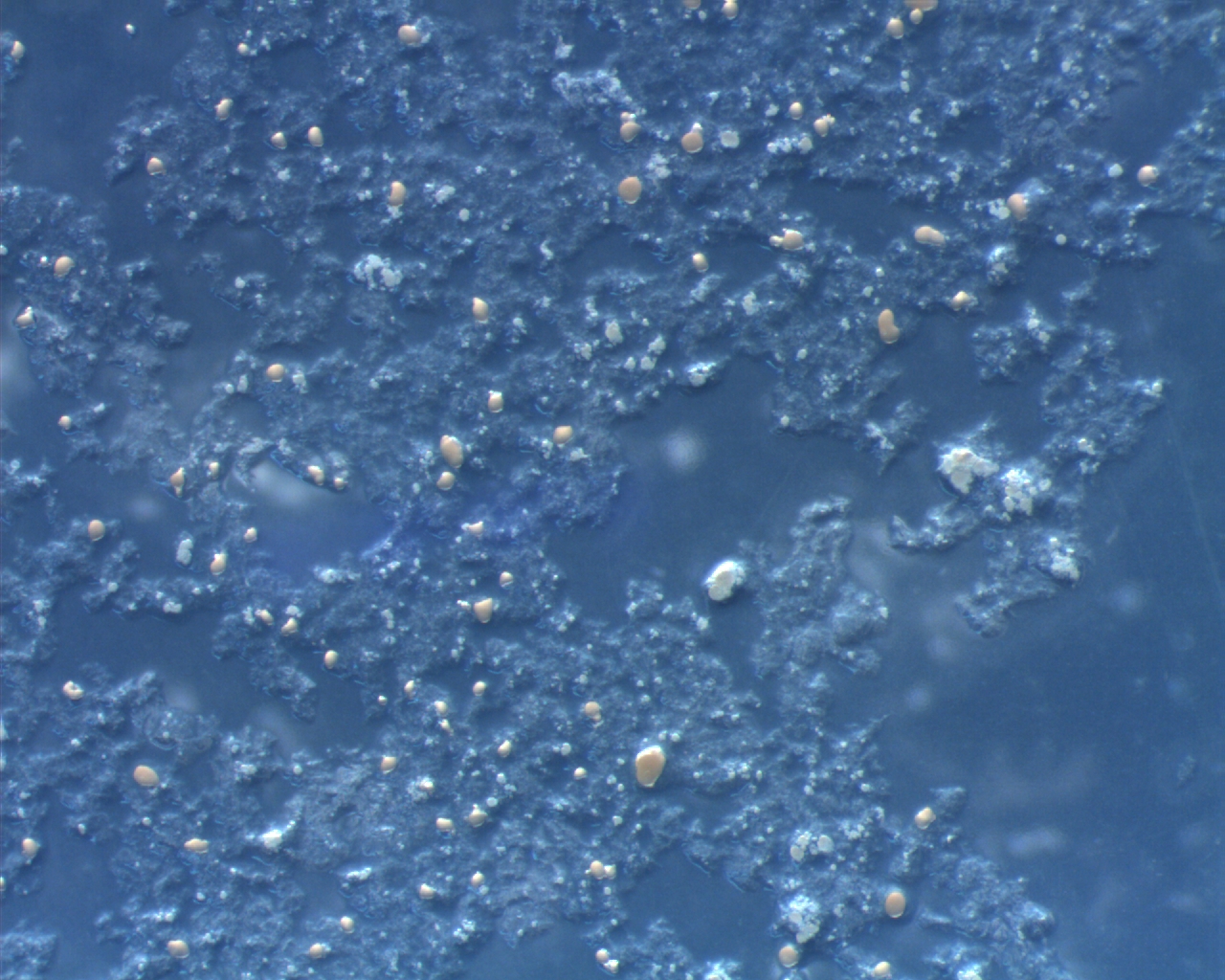

Supplement: Supplementary file 19 — Additional files 19: Uncropped image of M. stipitatus 26 h culture. [file 12864_2021_8051_MOESM19_ESM.jpg]

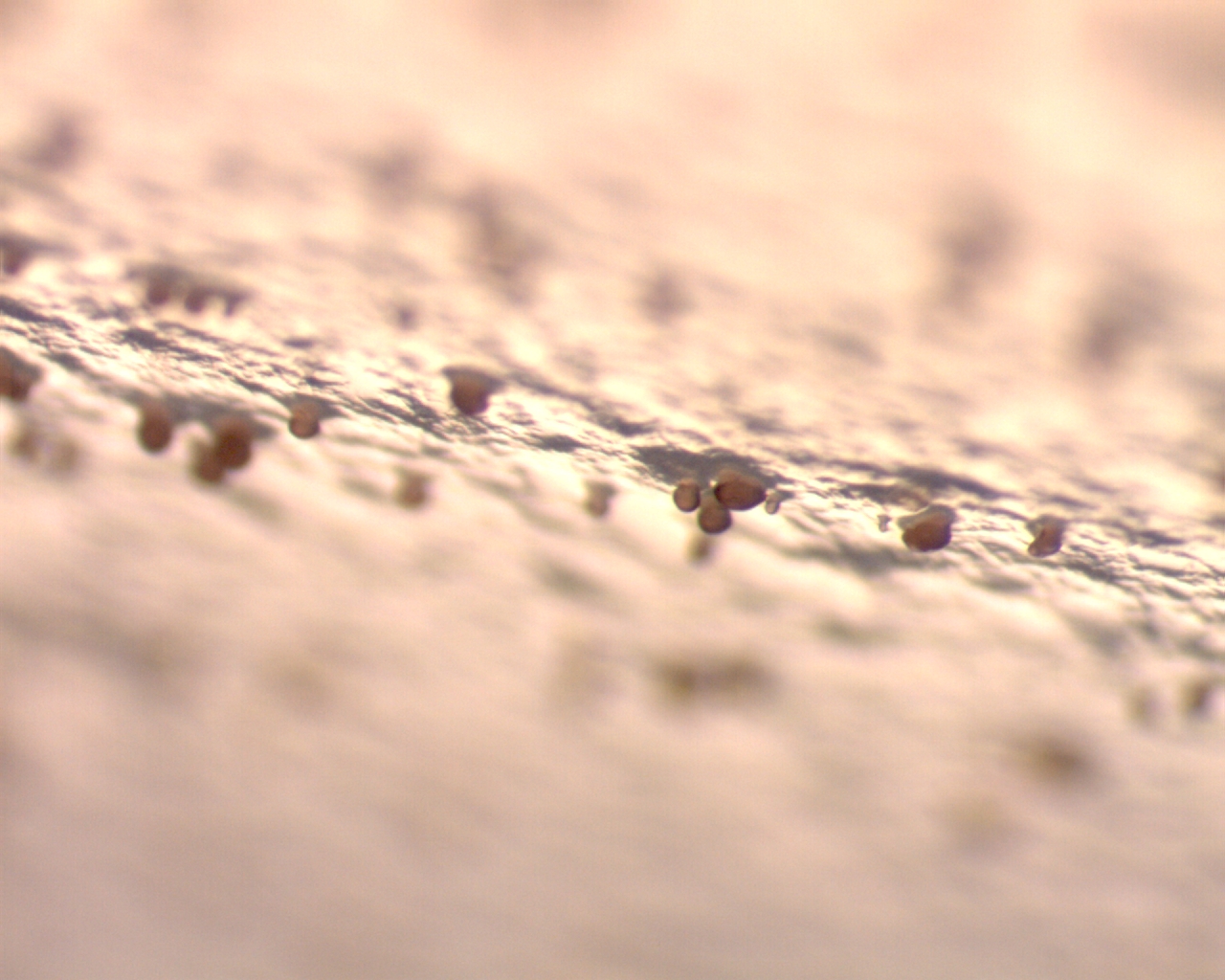

Supplement: Supplementary file 20 — Additional files 20: Uncropped image of M. stipitatus fruiting body side view. [file 12864_2021_8051_MOESM20_ESM.jpg]
