# Supplementary material for: Transcriptomic analysis of the Myxococcus xanthus FruA regulon, and comparative developmental transcriptomic analysis of two fruiting body forming species, Myxococcus xanthus and Myxococcus stipitatus
Source: BMC Genomics. 2021 Nov 1;22:784. doi: 10.1186/s12864-021-08051-w (PMC8561891; doi:10.1186/s12864-021-08051-w)
Supplement: Supplementary file 3 — Additional file 3: Supplemental table 1. Interpro domains that are enriched in each of the 4 K-means clusters for M. xanthus. [file 12864_2021_8051_MOESM3_ESM.docx]

**Additional table 1: Interpro domains that are enriched in each of the 4 K-means clusters for *M. xanthus***

| A | B | C | D |
| --- | --- | --- | --- |
| Phosphopantetheine attachment site | Pyridoxal-dependent decarboxylase conserved domain | Histidine kinase-, DNA gyrase B-, and HSP90-like ATPase | Response regulator receiver domain |
| Acyl transferase domain | Histidine kinase-, DNA gyrase B-, and HSP90-like ATPase | Response regulator receiver domain | Alpha amylase, catalytic domain |
| KR domain | PAS fold | His Kinase A (phospho-acceptor) domain | Domain of unknown function DUF20 |
| Glutathione S-transferase, N-terminal domain | His Kinase A (phospho-acceptor) domain | CHAT domain | PLD-like domain |
| Polyketide synthase dehydratase | GAF domain | AMP-binding enzyme | Carbohydrate-binding module 48 (Isoamylase N-terminal domain) |
| Condensation domain | Spore Coat Protein U domain | Phosphopantetheine attachment site | Endonuclease/Exonuclease/phosphatase family |
| AMP-binding enzyme C-terminal domain | Glycosyl transferases group 1 | Condensation domain | GGDEF domain |
| Biotin-requiring enzyme | Methyltransferase domain | Gram-negative bacterial TonB protein C-terminal | GLTT repeat (6 copies) |
| Type IV pilin N-term methylation site GFxxxE | Domain of unknown function (DUF4215) | AMP-binding enzyme C-terminal domain | YtxH-like protein |
| Glutathione S-transferase, C-terminal domain | Domain of unknown function (DUF4150) | FecCD transport family | Domain of unknown function (DUF4476) |
| Luciferase-like monooxygenase | ThiF family | Putative zinc-finger | Ferritin-like domain |
| Prolyl oligopeptidase family | Rhodanese-like domain | Sigma-54 interaction domain | Binding-protein-dependent transport system inner membrane component |
|  | Cytochrome C oxidase, cbb3-type, subunit III | Peptidase C39 family | Bacterial sugar transferase |
|  | Glycosyl transferase 4-like domain | Gene 25-like lysozyme | Virulence factor BrkB |
|  |  | HmuY protein | Uncharacterised protein family (UPF0104) |
